# Immunoprofiling of *Chlamydia trachomatis* using whole-proteome microarrays generated by on-chip *in situ* expression

**DOI:** 10.1038/s41598-018-25918-3

**Published:** 2018-05-14

**Authors:** Katrin Hufnagel, Smiths Lueong, Martina Willhauck-Fleckenstein, Agnes Hotz-Wagenblatt, Beiping Miao, Andrea Bauer, Angelika Michel, Julia Butt, Michael Pawlita, Jörg D. Hoheisel, Tim Waterboer

**Affiliations:** 1 Division of Molecular Diagnostics of Oncogenic Infections (F020), German Cancer Research Center (DKFZ), Heidelberg, Germany; 2 Faculty of Biosciences, Heidelberg University, Heidelberg, Germany; 3 Genomics Proteomics Core Facility HUSAR Bioinformatics Lab, German Cancer Research Center (DKFZ), Heidelberg, Germany; 4 Division of Functional Genome Analysis (B070), German Cancer Research Center (DKFZ), Heidelberg, Germany

## Abstract

Using *Chlamydia trachomatis* (Ct) as a complex model organism, we describe a method to generate bacterial whole-proteome microarrays using cell-free, on-chip protein expression. Expression constructs were generated by two successive PCRs directly from bacterial genomic DNA. Bacterial proteins expressed on microarrays display antigenic epitopes, thereby providing an efficient method for immunoprofiling of patients and allowing *de novo* identification of disease-related serum antibodies. Through comparison of antibody reactivity patterns, we newly identified antigens recognized by known Ct-seropositive samples, and antigens reacting only with samples from cervical cancer (CxCa) patients. Large-scale validation experiments using high-throughput suspension bead array serology confirmed their significance as markers for either general Ct infection or CxCa, supporting an association of Ct infection with CxCa. In conclusion, we introduce a method for generation of fast and efficient proteome immunoassays which can be easily adapted for other microorganisms in all areas of infection research.

## Introduction

*Chlamydia trachomatis* (Ct) is the globally leading cause of bacterial sexually transmitted infections (STI) with an estimated 131 million new cases of genital Ct infections per year. Symptoms of acute infection include e.g. painful urination, and urethral or vaginal discharge, but the majority of infections are asymptomatic. If untreated, chlamydia can give rise to chronic infection and sequelae that include pelvic inflammatory disease, chronic pelvic pain, ectopic pregnancy and tubal factor infertility^1^.

Ct is an obligate intracellular bacterium. During infection, the Ct infectious particle (elementary body, EB) invades epithelial cells of the genital tract via induced phagocytosis. It thereby generates a cytoplasmic inclusion where EB differentiate into non-infectious but metabolically active reticulate bodies (RB) that can undergo rapid replication^2^. During the acute infection cycle, RBs re-differentiate to EBs eventually exiting the infected cells either by a packaged release mechanism, or by cell lysis, and infect new target cells^3^. However, Ct can also enter a persistent infection state where RBs do not replicate, but persist as enlarged bodies in the host cell^4^. Persistent infection in women can result in chronic inflammation of the lower and upper genital tract that may be diagnosed as cervicitis or pelvic inflammatory disease (PID) which in turn can lead to chronic pelvic pain, tubal factor infertility, or ectopic pregnancy^4,5^. The complex molecular processes underlying both acute and persistent infections are mirrored by specific bacterial protein expression patterns^6^. However, most of these are only poorly, or not at all understood.

Although persistent infection with high-risk HPV types is a known prerequisite for cervical cancer (CxCa) development^7^, Ct has been discussed as co-factor in CxCa development, based on its biological features such as induction of inflammation, evasion of cell mediated immunity, inhibition of apoptosis, and involvement in DNA damage and genetic instability^8^. Additionally, large seroepidemiological studies have reported significant associations between Ct seropositivity and CxCa^9–12^.

Several serological assays have been developed to study the overall population prevalence of Ct infection as well as its associations with CxCa^9–12^ and the eye disorder trachoma^13–15^. However, most existing assays have only utilized a very small fraction of the almost 900 open reading frames (ORFs) encoded in the Ct genome. Antigen selection for immunoassays is usually based on prior knowledge about antigenic properties of the pathogen’s proteins, and thus restricted to few selected antigens. However, *de novo* identification of antigens distinguishing e.g. infected from non-infected individuals, or infected cancer cases from disease-free infected individuals is challenging for poorly studied pathogens, especially for bacteria due to their large number of encoded proteins.

Protein microarrays are excellent tools to identify disease-associated antibody reactivity patterns since they possess high density capacity and allow the simultaneous detection of antibodies to a large variety of antigens, up to an entire bacterial proteome. Previously published microarrays displaying whole proteomes of *Plasmodium falciparum*, *Leptospira interrogans* and *Bartonella henselae* were produced by performing PCRs for all genes of interest, followed by restriction digestion and cloning of PCR products into expression vectors, and subsequent transformation into *Escherichia coli (E. coli)*. After amplification of bacterial cultures, plasmids were purified and *in vitro* transcribed and translated. The resulting proteins were purified and printed on solid supports^16–18^. Following this method, Wang *et al*.^19^ described a genome-wide Ct microarray in ELISA microtiter plates. These approaches are extremely time-consuming, resource-intensive, and require large sample volumes. In order to eliminate the need for cloning, expressing, purifying and immobilizing the proteins individually, *in situ* protein array production strategies have been developed allowing proteins to be synthesized directly on the microarray surface using cell-free expression systems^20–22^. Angenendt *et al*.^23^ have developed the multiple spotting technique (MIST), where individually cloned expression vectors or PCR products are transferred onto microarray slides in a first spotting step. Subsequently, a cell-free transcription and translation mixture is spotted directly on top of the first spot in a second spotting step. As each synthesis is performed in few nanoliters, reagent consumption is low. Synthesis of each protein occurs in an individual droplet on the planar surface, minimizing the risk of contamination; also, no background is generated between the protein spots. Proteins binding to the solid support do not require capturing agents such as antibodies. Previous studies have shown that most proteins can be produced in full-length, and very many fold into a functionally active conformation^23,24^.

Therefore, it is highly desirable to develop an assay that circumvents individual cloning, expression and purification of hundreds or thousands of ORFs, in combination with the advantages of a slide-based microarray with regard to reagent and sample consumption.

Using Ct as a complex model organism, we describe a novel method to perform proteome immunoassays (PIA). Our method to produce bacterial whole-proteome microarrays is based on the combination of MIST and cell-free, on-chip protein expression based on expression constructs generated by two successive PCRs directly from bacterial genomic DNA. PIA bypasses both the generation of expression vectors, and purification and printing of proteins onto microarrays. Bacterial proteins expressed on the microarray can be recognized by serum antibodies, thereby providing an efficient method for immunoprofiling of patient samples which allows the *de novo* identification of disease-related antigens. By this approach, we provide data supporting an association of Ct infection with CxCa, and introduce a method for generation of fast and efficient proteome immunoassays which can be easily adapted to other microorganisms in all areas of infection research as well as e.g. autoantibody screening and epitope mapping.

## Results

### *In situ* protein expression

**Figure 1:**
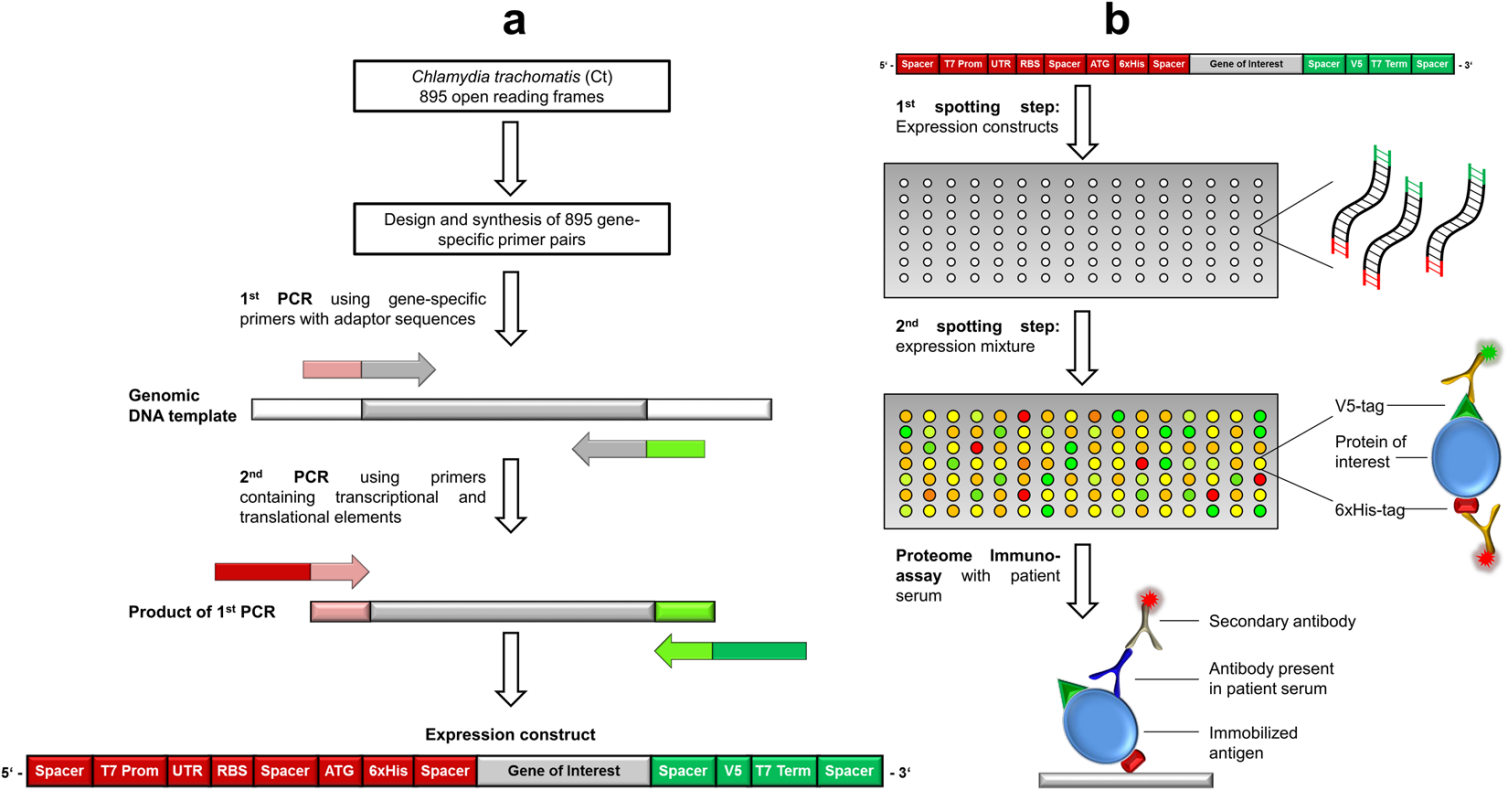
Generation of Ct whole-proteome microarrays. (**a**) In order to generate expression constructs, gene specific primer pairs were designed for all 895 Ct ORFs, including adaptor sequences common to all forward (pink) and reverse primers (light green). Using genomic Ct DNA as template, all 895 genes were amplified, resulting in PCR products carrying the gene of interest (GOI) flanked by two adaptor sequences. These products served as templates for a second PCR, using common primers for all Ct genes. These primers contain transcriptional and translational elements, as well as sequences complementary to the adaptor sequences. After the second PCR, genes were therefore flanked by spacer sequences, regulatory sequences (T7 Promoter, untranslated region (UTR), ribosome binding site (RBS), start codon (ATG), T7 Terminator) and N-terminal 6xHis and C-terminal V5 fusion tags (red: sequences included in the forward primer; green: sequences included in the reverse primer). (**b**) The expression constructs were transferred onto Ni-NTA coated slides in a first spotting step. Subsequently, the expression mixture was spotted directly on top of the first spots, and proteins were expressed on the slide during incubation in a humidified environment. Efficiency of protein expression was determined using fluorescence-conjugated antibodies directed against the terminal fusion tags. Proteome Immunoassays were performed by incubation of serum on whole-proteome arrays. Antibodies present in the serum were able to bind to the immobilized antigens on the array, and quantified using a fluorescence-conjugated anti-human secondary antibody.

The first step in order to create Ct whole-proteome microarrays was the generation of expression constructs by two successive PCRs for cell-free on-chip expression (Fig. 1a). The first PCR was performed using genomic Ct DNA as template and gene specific primer pairs for all 895 ORFs (listed in Supplementary Table S1). In addition to the 895 coding genes of *Chlamydia trachomatis* D/UW-3/Cx, the arrays contained the major outer membrane proteins (MOMP) of Ct serovars A and L2 and of *Chlamydophila pneumoniae* (Cp) to test for serovar specificity as well as cross-reactivity. To all gene specific primers, a common adaptor sequence was added. A second PCR was performed using the product of the first PCR as template and primers that consisted of all sequence features necessary for transcription and translation, sequences encoding for N-terminal 6xHis and C-terminal V5 tags as well as sequences complementary to the respective adaptors of the first primers. Thereby, the same primer pair could be used for all second PCR amplifications. All 898 genes were successfully amplified by the two successive PCRs. The products of each PCR were analyzed by agarose gel electrophoresis, and for each ORF correct fragment length was verified.

**Figure 2:**
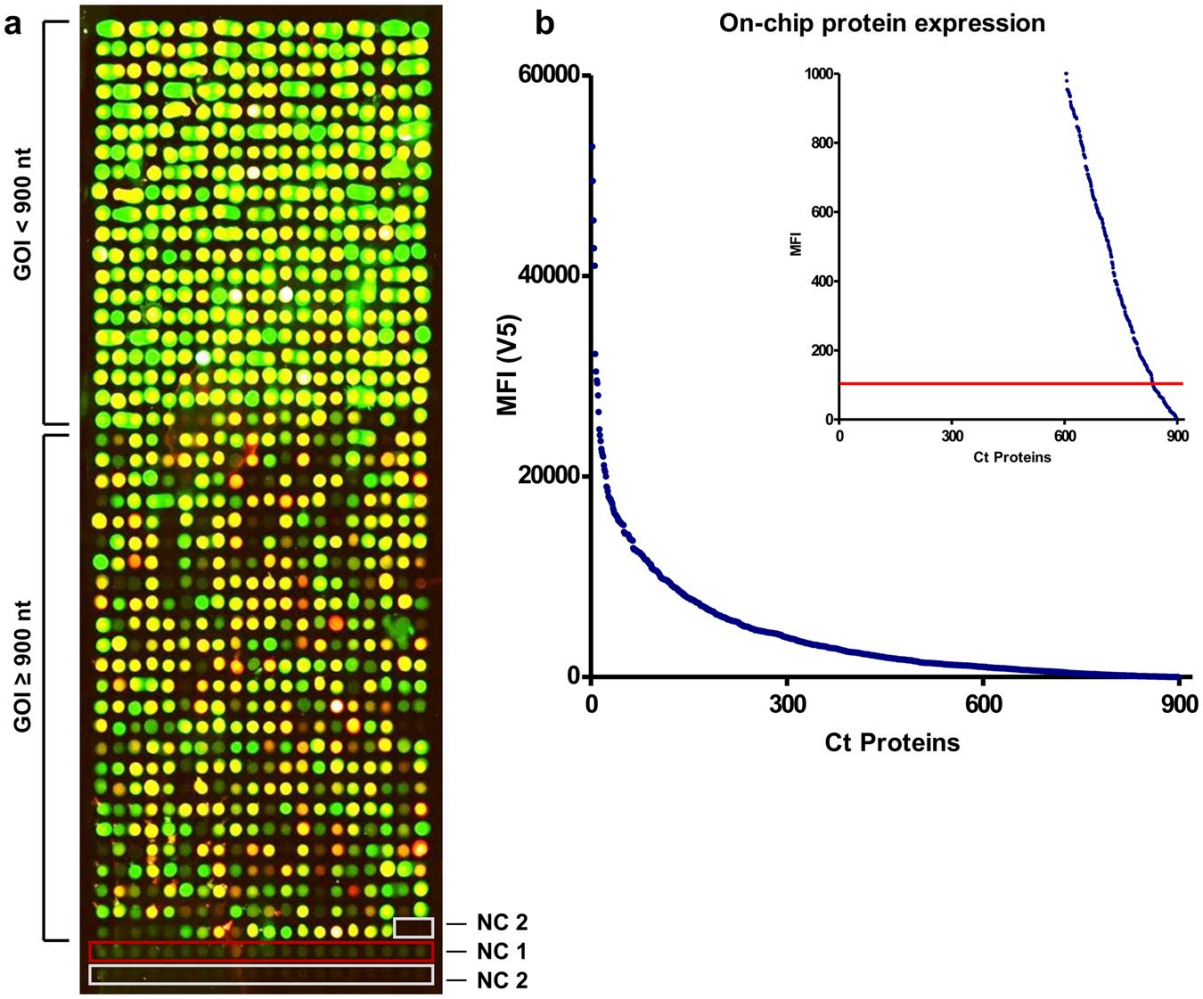
Microarray displaying the whole-proteome of Ct. (**a**) Results of the determination of on-chip protein expression using antibody staining against the terminal tags (green signal: anti-V5 antibody; red signal: anti-His antibody; yellow signal: overlay of both signals). All 898 proteins were spotted onto one array. The last 22 spots indicated by red and white frames represent the negative controls NC1 and NC2, respectively. The upper part of the array comprised genes of interest (GOI) with a DNA template length of up to 900 bp. (**b**) Final MFI values obtained by the anti-V5 antibody were sorted in descending order and plotted against the overall number of Ct proteins. The calculated cut-off (100 MFI) is represented by a red line, indicating that less than 10% of all proteins are below the cut-off and are therefore considered not to be expressed in full length.

The product of the second PCR was used as expression construct and was transferred onto microarray slides. A cell-free expression extract was spotted onto the first DNA template-containing spot and proteins were expressed directly on the microarray slide (Fig. 1b). On the whole-proteome microarray representing 898 proteins and two types of negative controls (NC1/2), on-chip protein expression was determined by antibody staining against the N-terminal 6xHis and the C-terminal V5 tag (Fig. 2a). For NC1, PCR products from both successive PCRs using water instead of DNA as template was spotted as expression construct. For NC2, no expression construct but only the expression mixture was spotted onto the microarray. A protein was considered to be expressed if the signal generated by labeling either the 6xHis or the V5 tag was higher than the mean plus five standard deviations of 20 NC1 replicates. Based on N- or C-terminal detection only, 818 (91.1%) and 837 (93.2%) of all genes were successfully expressed, and 867 genes (96.5%) showed either C- or N-terminal expression above cut-offs. N- and C-terminal expression signals are included in Supplementary Table S1.

For ORFs with a length of up to 900 bp, expression was 100% successful, and all proteins generated C-terminal signals indicating full-length expression. ORFs longer than 900 bp generated lower signal intensities overall, and showed in some cases only N-terminal expression signals. MFI values for the anti-V5 signals are illustrated in Fig. 2b.

### Proteome immunoassays (PIA)

**Figure 3:**
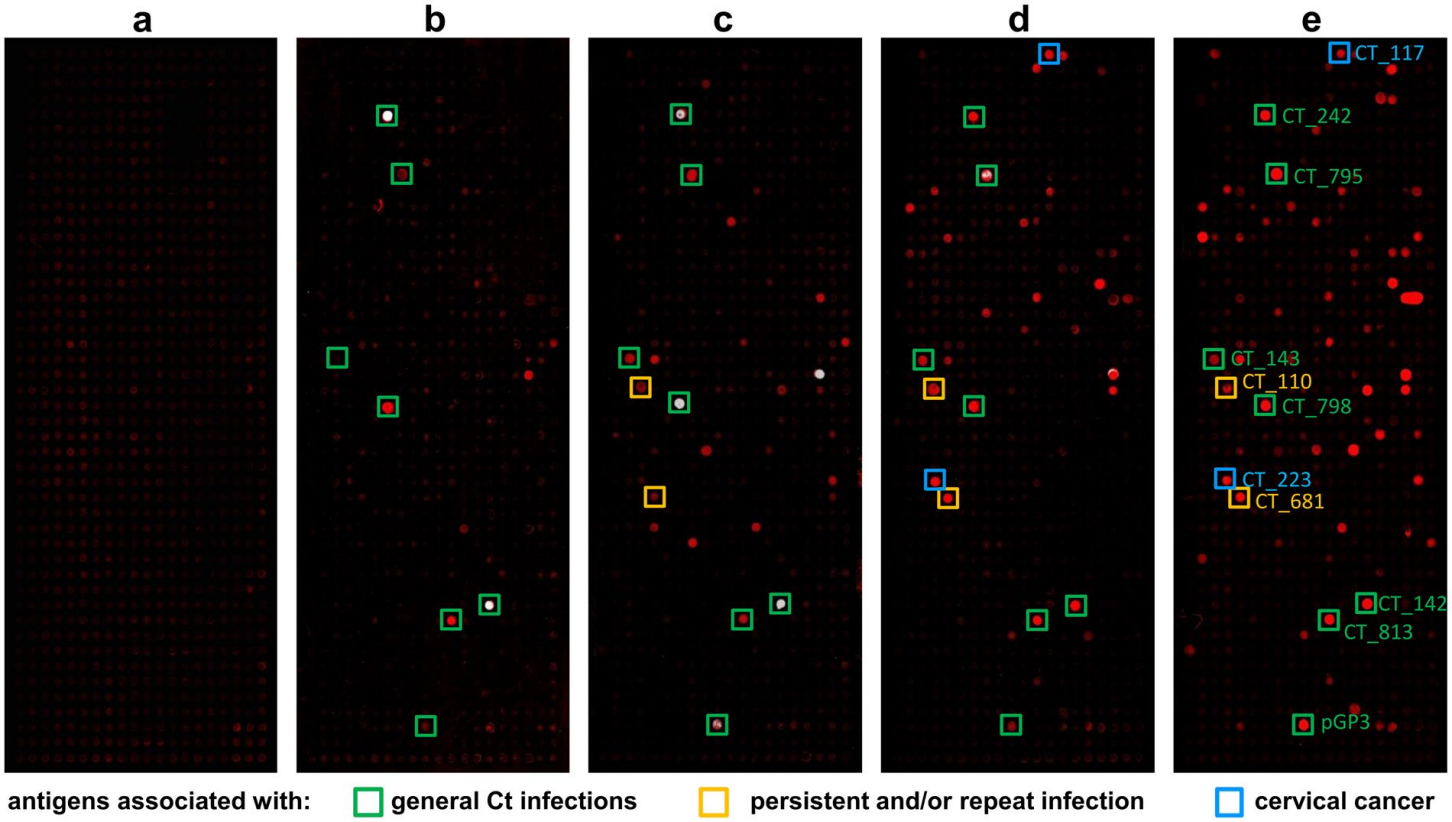
Proteome Immunoassays (PIA) using pools of five sera. In total 897 Ct proteins and one Cp protein were spotted on one array. (**a**) No signal was obtained with a pool of five sera from Ct-uninfected women. (**b**) Result of PIA with a pool of 5 sera from cancer-free women infected with Ct and <25 years of age. (**c**) Same as b, but women >40 years of age. (**d**,**e**) Results of PIAs with two different pools of samples from Ct-infected cervical cancer patients. Examples for antigens associated with general Ct infections (**b**–**e**), potentially persistent infections (**c**–**e**) and cervical cancer (**d**,**e**) are highlighted with green, yellow, and blue boxes, respectively.

Human sera were pooled according to their pre-determined Ct serostatus and Ct DNA positivity, their age and CxCa case-control status. Since Ct infections are usually acquired at young age^25^, we hypothesized that young women are more likely to suffer from acute infections while older women have a higher probability of having developed persistent infections. Therefore, we additionally grouped the Ct-infected women according to their age. As a pilot study, we incubated 22 pools of each five sera on the whole-proteome array: two Ct-uninfected, twelve Ct-infected cancer-free, and eight Ct-infected CxCa pools. Binding of serum antibodies to antigens immobilized on the microarray was detected by a secondary fluorescence-conjugated anti-human antibody. Typical results of PIAs are shown in Fig. 3. Sera from uninfected women (negative for both genital Ct DNA and pre-determined overall Ct antibody status) did not show positive signals with any of the Ct proteins (Fig. 3a), while all serum pools of Ct-infected women revealed positive signals (Fig. 3b–e). Besides the identification of novel antigens, we were able to confirm known immunogenic Ct proteins that are already used in many serological assays such as the plasmid encoded pGP3 and CT_681 (major outer membrane protein, MOMP). Comparison of the antibody reactivity patterns indicated that there were antigens which reacted with all Ct seropositive pools but also antigens which reacted only with serum pools from cancer patients. Interestingly, we observed some antigens reacting only with pools of sera from women of higher age, possibly indicating persistent Ct infections. None of the Ct positive sera analyzed on the whole-proteome array showed reactivity with Cp MOMP, and only few sera showed low reactivity with the MOMP of serovars A and L2. Supplementary Figure S2 illustrates the results of all 22 PIAs.

In order to exclude a concordance between PIA signals and signals obtained during determination of on-chip protein expression both signals were compared by linear regression. Correlation coefficients below 0.05 across 20 Ct seropositive serum pools revealed no correlation between protein expression levels and immunoassay signals. In fact, some Ct proteins with very low expression signals revealed high MFI with some serum pools. Therefore, immunoassay signals were not normalized for their respective expression signals.

**Figure 4:**
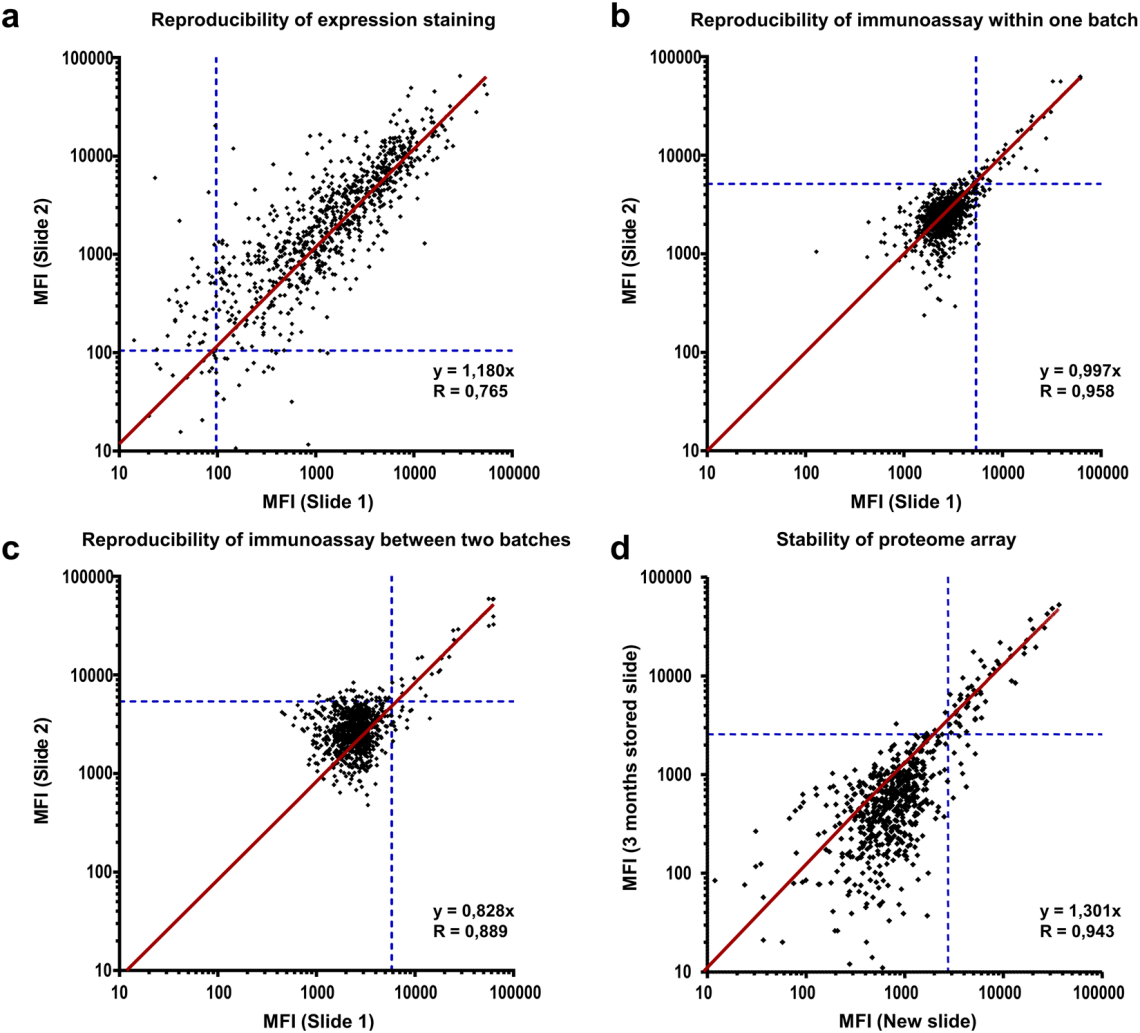
Reproducibility and stability of analyses on Ct whole-proteome microarrays. Correlation of MFI values obtained in two independent experiments was determined by linear regression analysis (forced through the origin) and is expressed by the slope and Pearson’s correlation coefficient (r). Cut-offs are highlighted by dashed blue lines. (**a**) Expression staining using antibodies against fusion tags of the proteins was replicated on two slides from the same production batch. Each dot represents one Ct protein with anti-V5 MFI values from two independent experiments. (**b**) Repetition of an immunoassay for the same pool of five sera on two slides produced within the same batch. (**c**) Same as b, with slides from two different production batches (same pool as in panel b). (**d**) Repetition of an immunoassay for the same pool of five sera on two slides (different pool from panels b and c). One Immunoassay was performed shortly after production of the microarray and one slide was stored for a time period of 3 months before the assay was performed.

Both protein expression staining and PIA showed overall good reproducibility in independent experiments (Fig. 4). A correlation coefficient of 0.77 revealed good reproducibility for the expression staining (Fig. 4a). Reproducibility of immunoassays was particularly high (r = 0.96) when performed with microarrays produced in a single production batch (Fig. 4b). Variation was only slightly higher when the microarrays were produced in different batches (r = 0.89, Fig. 4c).

In order to test the stability of the proteome microarrays, slides were stored over a time period of 3 months. When comparing PIA performed on freshly produced and stored microarrays, slightly higher signal intensities were observed on the fresh microarray in the area of lower MFIs. This might be explained by an overall higher background (NC1) signal after storage of the slides. However, a correlation coefficient of 0.94 indicated high reproducibility. After applying cut-offs, 54 antigens were concordantly positive on both slides, and 833 were concordantly negative. Three and 8 antigens were only identified on the stored and fresh slides, respectively, corresponding to an overall agreement of 98.8% and a Cohen’s kappa of 0.90 (95%CI 0.84–0.96). Among highly immunogenic antigens (signal stronger than 10 standard deviations above background), the concordance between fresh and stored slide was 100%.

**Figure 5:**
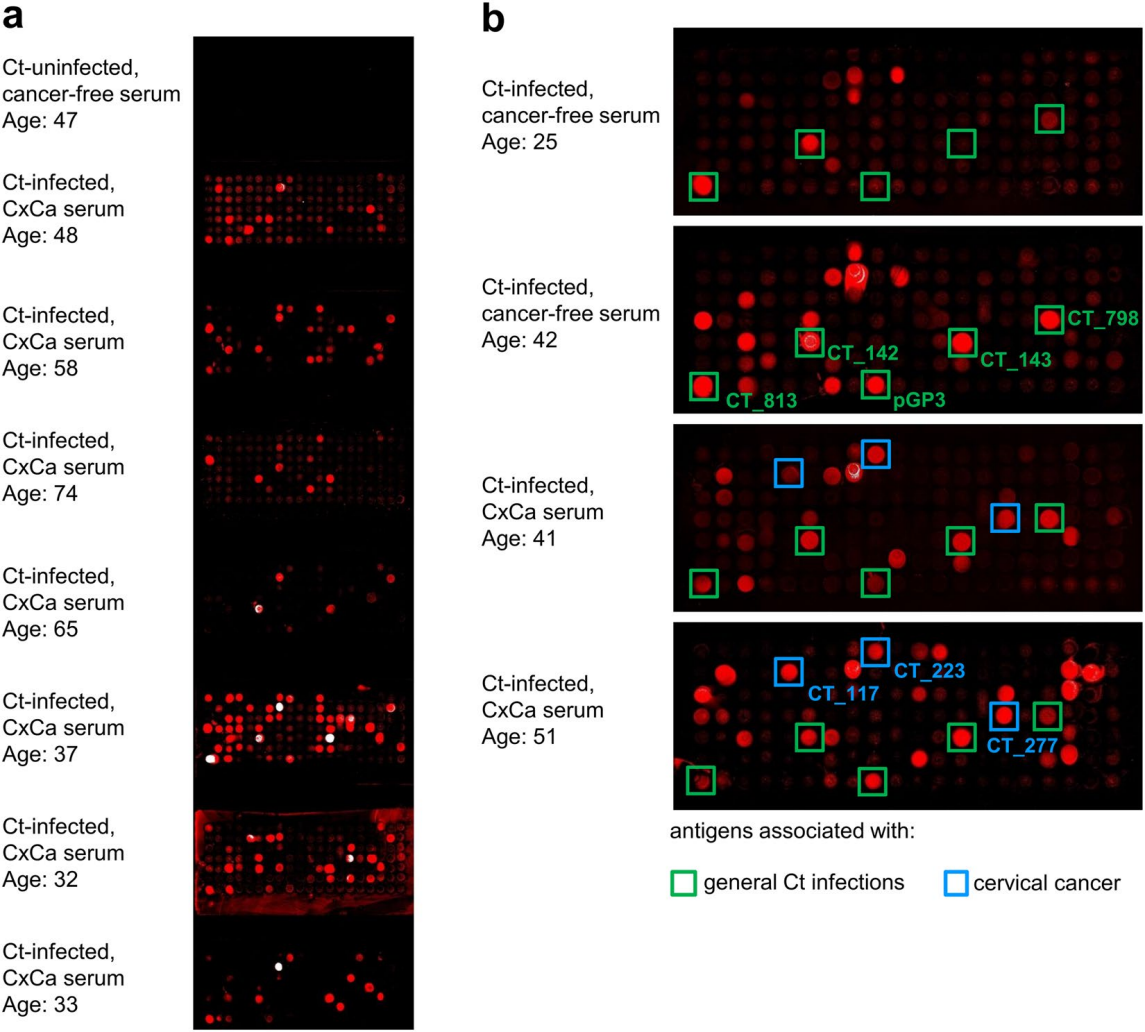
Antibody patterns in individual sera with 130 selected Ct antigens. (**a**) Each microarray comprised 8 blocks and each block contained the same 130 Ct antigens and 10 negative controls (NC1). Individual blocks were incubated with serum from a Ct-uninfected woman (topmost block), and seven Ct-infected cervical cancer patients. (**b**) Enlarged view of antibody reactivity patterns in single sera from Ct-infected women, the top two being cancer-free and the bottom two with cervical cancer. Boxed spots highlight potential markers for general Ct infection (green) and cervical cancer (blue).

Based on the results of the whole-proteome array, we considered 130 antigens to be potentially informative in discriminating antibody patterns. These were selected for further analyses with individual sera, either because they reacted with at least two of the 22 tested pools, or they generated a particularly strong signal with only one pool (signal stronger than 10 standard deviations above the mean of 20 NC1 replicates). These antigens were expressed on microarrays containing eight blocks separated by frames, with each block containing 140 spots (130 selected antigens and 10 negative controls (NC1)). This setup allowed incubating eight sera on one array. A typical result is shown in Fig. 5a.

The topmost block (Fig. 5a) was incubated with serum from a Ct-uninfected woman which gave no positive signal, as expected. In contrast, on the other seven blocks, strong signals were observed after incubating sera from Ct-infected CxCa patients. Comparison of antibody reactivity patterns (Fig. 5b) revealed antigens which reacted with all tested samples from known Ct-infected women (n = 150), as well as antigens reacting only with samples from cancer patients (n = 40).

### Validation using multiplex serology

In PIA analysis of single sera, we identified previously known highly prevalent antigens such as MOMP (CT_681) and pGP3 as markers for Ct infection. As our focus was *de novo* biomarker discovery, we focused on several newly identified promising antigens to detect general Ct infections: two hypothetical proteins (CT_142, CT_143), a glycogen synthase (CT_798) and an inclusion membrane protein (CT_813). These four antigens reacted with >90% of all tested known Ct-infected samples. In addition, two antigens which reacted with most of the tested samples from CxCa patients but not with cancer-free infected individuals were selected as potentially CxCa associated antigens: inclusion membrane proteins CT_117 and CT_223. All six antigens were validated using low-density, high-throughput multiplex serology.

This method is a bead-based suspension array technology capable of efficiently analyzing thousands of serum samples for antibodies to a limited number of antigens. The higher throughput of multiplex serology improves statistical power, thus allowing to investigate the ability of whole-proteome microarrays to detect novel infection and disease associated antibody reactivity patterns. Antigens were expressed as recombinant GST-fusion proteins and loaded on glutathione-casein coupled spectrally distinct fluorescence-labeled polystyrene beads. Antigen-loaded beads were mixed and incubated with sera. Serum antibodies bound to antigen-loaded beads were quantified using a labeled secondary antibody^26^.

Validation sera were selected from a Mongolian population-based cross-sectional HPV prevalence study^27^ and from a series of 96 histologically confirmed Mongolian CxCa cases^28^. Ct DNA status in cervical liquid based cytology specimens was determined by PCR^29^.

**Figure 6:**
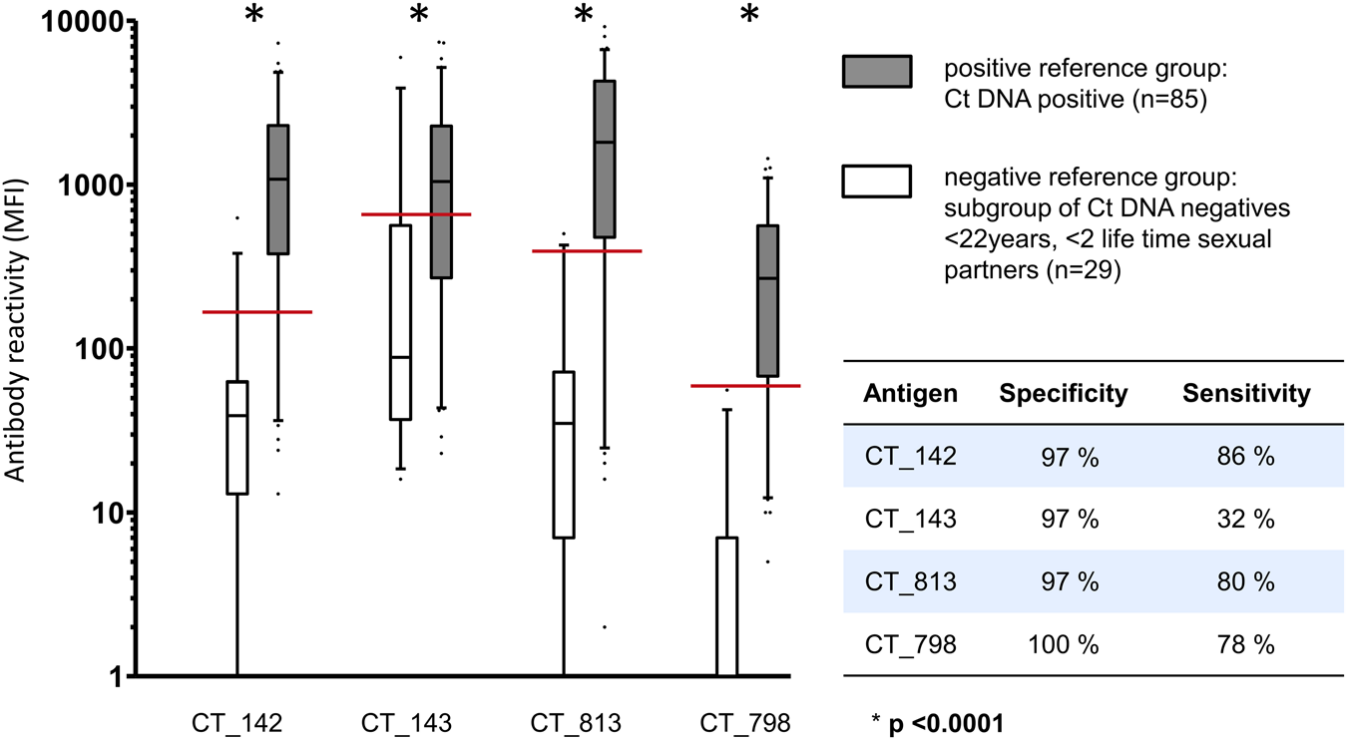
Performance of *de novo* identified antigens in multiplex serology. Two Ct infection reference groups (grey, positive, and white, negative) were analyzed with four antigens representing possible new serological Ct infection markers. On the y-axis, MFI are plotted on a logarithmic scale, and distribution of reactivities for each group and antigen is displayed as box plot. Cut-off values were determined by Receiver Operating Characteristic (ROC) analysis and are shown as red lines resulting in specificity and sensitivity estimates shown in the inserted table.

Two reference groups for validation of general Ct infection markers were analyzed: a positive reference group of Ct DNA positive women (n = 85), and a negative reference group comprising a subgroup (n = 29) of Ct DNA negative women less than 22 years old and with less than two life time sexual partners. All four antigens significantly distinguished the two reference groups (Fig. 6, all p < 0.0001). ROC curve analysis was used to determine cut-offs for each antigen, yielding specificities ≥97% for all antigens (Fig. 6). Three antigens (CT_142, CT_813 and CT_798) showed sensitivities ≥78% and were therefore considered to be promising antigens to serologically detect Ct infections (Fig. 6). In total, we analyzed sera from 985 Mongolian women by multiplex serology using our newly identified antigens in comparison to an already known immunogenic Ct antigen, pGP3. Supplementary Table S3 describes good assay concordance between pGP3 and the novel four biomarkers with kappa values between 0.68 and 0.83.

**Figure 7:**
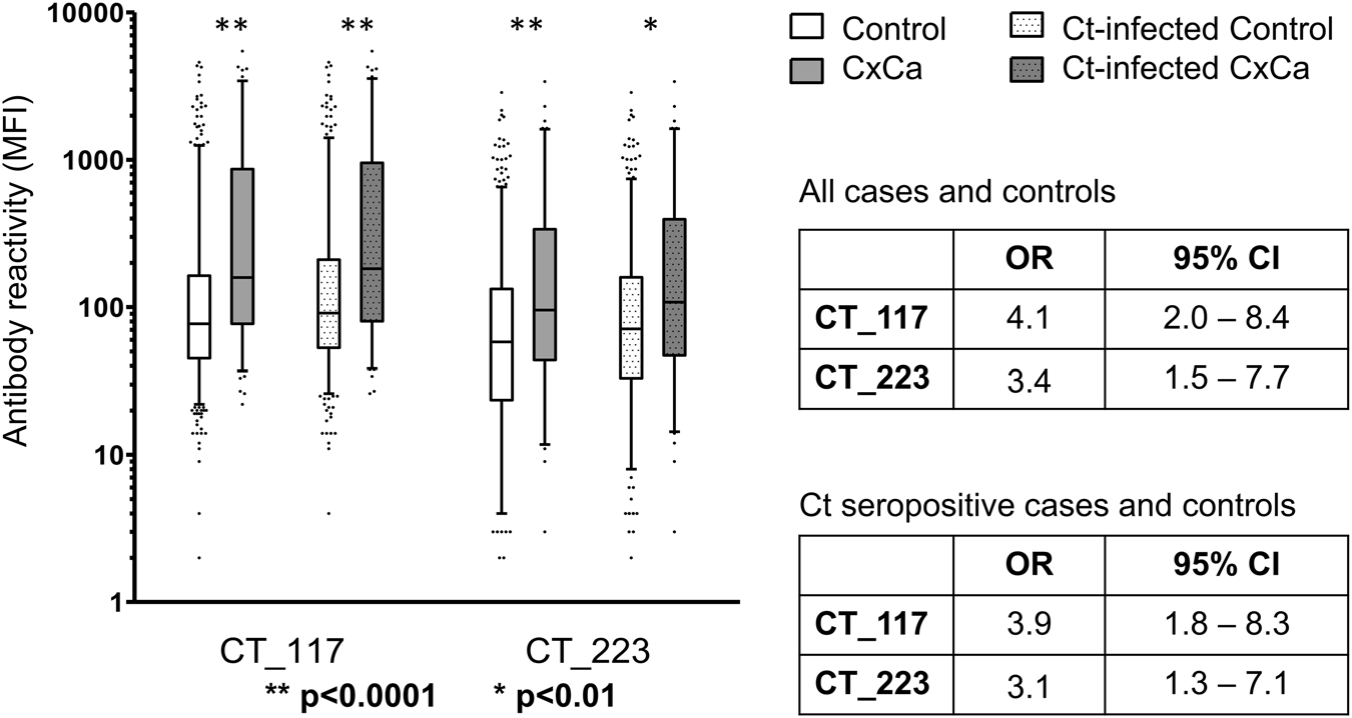
Increased antibody reactivities and risk of CxCa for serum antibodies to Ct antigens CT_117 and CT_223. Seropositivity for each of the two proteins was significantly associated with elevated CxCa risk when comparing all CxCa cases and all controls as well as when restricting the risk assessment to Ct-infected individuals among both cases and controls. OR, odds ratio; CI, confidence interval.

We further compared the reactivity of the two potentially CxCa-associated antigens in 96 histologically confirmed Mongolian CxCa cases and 520 controls from the population study covering the same age range as the CxCa cases (Fig. 7). Antibody reactivities to both CT_177 and CT_223 were significantly elevated in CxCa cases compared to controls (comparing all cases and controls, p < 0.0001 for both proteins; among Ct-infected cases and controls, p < 0.0001 for CT_177 and p < 0.01 for CT_223). An increased risk of CxCa was confirmed in the presence of serum antibodies to both antigens (CT_177: odds ratio (OR) 4.1, 95% confidence interval (CI) 2.0–8.4; CT_223: OR 3.4, 95%CI 1.5–7.7). Furthermore, when restricting the cervical cancer risk assessment to Ct-infected individuals among both cases and controls, seropositivity for each of the two proteins was significantly associated with elevated cervical cancer risk (CT_177: OR 3.9, 95%CI 1.8–8.3; CT_223: OR 3.1, 95%CI 1.3–7.1). These data indicate the potential of these newly identified antigens as serological risk markers for Ct-associated cervical cancer.

## Discussion

### Technical development of whole-proteome arrays

We have developed a novel method to express *in situ* the entire proteome of Ct as individual proteins on microarrays, and demonstrated the potential of these arrays to differentiate infection- and disease-associated antibody responses in patient sera. DNA templates for protein expression were obtained from bacterial genomic DNA by two successive PCRs for all bacterial ORFs. Thereby, we circumvent the need to clone all ORFs into plasmid expression vectors as well as to transform *E. coli* cells and to replicate and purify these vectors. Using MIST, we generated microarrays by cell-free on-chip protein expression with minimal amounts of DNA templates and expression mixture instead of printing purified proteins onto the arrays.

We were able to successfully express more than 96% of all Ct proteins. ORFs of >900 bp length generated lower signal intensities overall, and in some cases showed no full-length expression. Nonetheless, there was no correlation between protein expression levels and antibody reactivity, thereby indicating that protein expression levels do not influence PIA signals and even a small amount of protein is enough to detect serum antibodies. Analyses with pooled patient sera yielded 130 antigens that were informative for assessing either Ct infection or CxCa status, representing 14% of the complete Ct proteome. Although 14 of them showed only N-terminal expression in the whole-proteome approach, they were still recognized by antibodies from sera of infected individuals. These proteins might be the result of a premature translation stop and thus could be C-terminally truncated, while the epitope recognized by the serum antibodies might be located in the N-terminal region of the protein. The C-terminal V5 tag epitope also might have been masked and therefore inaccessible due to protein folding. Thus, even with undetected C-terminal signal, the protein might have been expressed in full-length. Of the 130 selected antigens, 49 were hypothetical proteins with unknown function. However, 16 of the informative antigens were reported to be located either in the Chlamydia outer membrane or the inclusion membrane, thereby indicating that bacterial membrane proteins are expressed and epitopes of such can be recognized by serum antibodies.

Reproducibility of the newly developed array was evaluated by repeating expression stainings and immunoassays. Overall, reproducibility was high, and arrays could be stored for three months without significant loss in reactivity. In our hands, inter-batch variation seems to be mainly caused by inaccuracy of the Nanoplotter during microarray production (either during sample uptake or dispension), although this effect is challenging to quantify.

### Antigen identification

In order to identify Ct-infection specific antigens, sera from Ct-infected women as well as controls with no history of Ct infection were incubated onto the microarrays. As expected, we were not able to detect antibodies against any of the Ct proteins after incubating sera from uninfected controls on the whole-proteome array, as these women were never exposed to Ct. This documents the specificity of the infection-specific binding. In contrast, infected women showed evidence of serum antibodies binding to immobilized Ct antigens. No tested pool was reacting with the MOMP of Cp, indicating Ct specificity without cross-reactivity to a closely related bacterium.

Pools of sera from younger patients reacted with less antigens compared to pools of older patients. This may be based on repeated Ct exposure of the older individuals, as many antibodies can be regarded as cumulative exposure markers. Another explanation might be the chlamydial life cycle comprising both acute and persistent states of infection. Some Ct proteins might not be expressed during the acute infection but only after entering the persistent infection state.

During infection, Ct is manipulating and interacting with the host and thereby causing DNA damage, genetic instability, induction of inflammation and inhibition of apoptosis^8^. This may require expression of varying sets of Ct proteins which are presented to the host immune system during various stages of interaction between host and pathogen. Some antigens identified in our analysis were previously described to interact with host proteins or pathways^30–32^. In particular, proteins of the Ct inclusion membrane are exposed to the host cytosol and are reported to be involved in host-pathogen interactions^33^. They lack sequence similarities between each other, or to proteins of other pathogens. Consequently, they are very promising biomarkers to detect Ct infections or Ct-associated diseases without showing cross-reactivity to other pathogens^34^. During as well as after transformation into cancerous tissue, cell and tissue damage occurs frequently. In damaged tissue, the intracellular inclusions of Ct are therefore released from the host cell and extracellularly exposed to the host immune system. The CxCa associated antigens identified in this work are Ct proteins of the intracellular inclusion and are usually not exposed to the immune system in healthy tissue. This would also explain the overall higher immune response of CxCa patients compared to cancer-free infected individuals.

One potential limitation of this study is the preliminary algorithm that we used to select promising antigens for PIA analysis of single serum samples. While we aim at comprehensive bioinformatics analysis of whole-proteome data in the future, we employed a simple but effective approach for this proof of concept study, by lowering the threshold for antigen selection. Every antigen that reacted with two (or strongly with one) out of 22 serum pools was included in the smaller, single sample arrays, thus retaining a very high sensitivity in *de novo* antigen identification.

### Validation using multiplex serology

In total, we have analyzed six selected Ct antigens using multiplex serology, a method that has been successfully employed in more than 150 published epidemiological studies by now. We analyzed homologies between the identified promising Ct antigens and proteins of the closely related pathogen Cp. There was no significant similarity found between CT_813, CT_117 and CT_223 and any Cp protein, therefore we do not expect cross-reactivity for any of these antigens with antibodies against Cp antigens. For CT_142 and CT_143, a maximum identity of 35% to hypothetical proteins of Cp was found. CT_798 is a glycogen synthase and shows 53% sequence identity to two Cp glycogen synthases. Thus, we cannot exclude a certain degree of cross-reactivity for this marker. Another strength of our study is that we were able to validate initial results obtained with the whole-proteome microarray in a large scale, well characterized epidemiological study with robust statistical analysis. Previous seroepidemiologic investigations observed significant associations between Ct antibodies and CxCa with OR between 1.6 and 2.2^9–11^. We analyzed 96 CxCa cases and 520 controls, covering the same age range as the cases, for presence of antibodies to the newly identified CxCa-associated antigens. Serum antibodies to CT_117 and CT_223 were associated with a statistically significant 3- to 4-fold increased risk for CxCa after adjustment for confounders including high risk HPV. Therefore, proteins CT_117 and CT_223 might be promising biomarkers to not only discriminate between cancer-free Ct-infected individuals and Ct-infected CxCa patients, but also to contribute to quantifying the attributable fraction of Ct in CxCa development.

### Summary and outlook

The microarray platform and multiplex serology complement each other during the process of *de novo* antigen identification. While the planar microarray permits the screening of relatively few samples for informative antibodies to an entire bacterial proteome, multiplex serology allows screening thousands of serum samples for relatively few antigens. In 2010, Wang *et al*. identified 27 immunogenic Ct antigens which reacted with more than 50% of 99 analyzed Ct-infected human sera^19^. Using our whole-proteome approach, we were able to confirm 20 of these antigens. Sixteen of these antigens were also associated with general Ct infection in our study. Other Ct proteins such as CT_110 (GroEL, Hsp60) were associated with persistent infection in our study and were also identified by Wang *et al*. However, since they reacted with lower frequency (47% of all tested sera) in their study, Wang *et al*. did not include them into the list of 27 immunogenic Ct antigens. All four antigens for general infection that we validated with multiplex serology had been reported by Wang *et al*. as immunogenic Ct proteins, and we have shown good concordance with a validated pGP3 assay (Supplementary Table S3) indicating that microarrays are useful tools to identify antigens compared to established methods such as ELISA and multiplex serology. In future analyses, we will utilize the Ct whole-proteome microarray to identify disease-specific antibody responses for other Ct-associated diseases such as pelvic inflammatory disease, ectopic pregnancy and ovarian cancer and evaluate their potential to serve as prognostic biomarkers in large-scale prospective cohort studies.

In addition, the method we have developed to produce whole-proteome microarrays can easily be adapted to other microorganisms in all areas of infection research. We have already initiated generation of whole-proteome microarrays for *Helicobacter pylori*, and plan corresponding analyses for all eight human herpes viruses, to investigate the role of these pathogens in the development of different types of human diseases.

## Methods

### Whole-genome PCRs for all Ct genes

The genome of Ct serovar D consists of 895 open reading frames (ORFs) of which 887 are genomic and 8 are plasmid-encoded. We further included the major outer membrane proteins (MOMP) of Ct serovars A and L2 and the MOMP of *Chlamydophila pneumoniae* (Cp) to analyze potential cross-reactivity between different Ct serovars and the closely related bacterium Cp. In order to generate Ct whole-proteome arrays, two successive PCRs were performed for all ORFs. In the first PCR, gene-specific primers were used to amplify all ORFs, separately in three 384-well microtiter plates. The 895 primer pairs were designed by the DKFZ bioinformatics core facility Heidelberg Unix Sequence Analysis Resources (HUSAR) using the reference genome Ct D/UW-3/Cx^35^ and synthesized in 96-well plates (Biomers). For primer design, a Perl script was generated to calculate primers using the ORF table information text file and fasta sequences of the Ct genome. The primers were designed to bind at the beginning and the end of each ORF (without stop codon), and to yield a product in frame, with primer lengths between 16 and 24 bases. The melting temperatures (Tm) of possible primers of different length were calculated with Melttemp (http://www.biology.wustl.edu/gcg/melttemp.html). Because all PCR reactions were designed to have approximately the same melting/annealing temperatures, the best fitting primers within a range of either 45 °C to 55 °C or 55 °C to 65 °C were selected. If no suitable primer was found, primer sequences starting from positions one or two triplets inside the product were considered. With fuzznuc (EMBOSS, http://emboss.sourceforge.net/apps/cvs/emboss/apps/fuzznuc.html) the final primer pairs were checked for uniqueness of their sequence, and the product length was calculated. To all 5′-ends of the primers, a common 15 nt adaptor sequence was added (forward adaptor primer: 5′-ATGCACCAAACCCAA-3′; reverse adaptor primer: 5′-CGCACTGGCATCATC-3′). Amplification reactions (25 µl) were prepared using genomic Ct DNA as template (Ct strains D/UW-3/Cx, A/HAR-13, 434/Bu and Cp strain TWAR-183; obtained from the German Collection of Microorganisms and Cell Cultures, DSMZ) and Q5 High-Fidelity DNA Polymerase (NEB) following manufacturer’s instructions. PCRs were performed in 384-well thin wall microseal PCR plates (Bio-Rad) in groups based on the length and melting temperature of each ORF <900 bp, 900-3000 bp, >3000 bp). An initial 2 min denaturation step at 98 °C was followed by 35 cycles of amplification (DNA Engine DYAD Peltier Thermal Cycler; MJ Research). Each cycle comprised a denaturation step at 98 °C for 15 s, an annealing step between 48 °C and 52 °C (depending on the Tm of the primers) for 30 s and an elongation step at 72 °C between 1:30 and 3:00 min (depending on the length of the ORF) followed by a final elongation step for 10 min. Of this first PCR, 1 µl PCR product carrying the gene of interest flanked by two adaptor sequences was used as template for the second PCR. The second PCR was based on a pair of expression primers carrying all sequences necessary for transcription and translation (T7 Promoter, untranslated region (UTR), ribosome binding site (RBS), start codon (ATG), T7 Terminator), fusion peptide tags (N-terminal 6×-His and C-terminal V5 tags) and overhangs complementary to the adaptors of the gene specific primers (forward expression primer: 5′-GAAATTAATACGACTCACTATAGGGAGACCACAACGGTTTCCCTCTAGAAATAATTTTGTTTAAGAAGGAGATATACATATGCATCATCATCATCATCATATGCACCAAACCCAA-3′; reverse expression primer: 5′-CTGGAATTCGCCCTTTTATTACGTAGAATCGAGACCGAGGAGAGGGTTAGGGATAGGCTTACCCGCACTGGCATCATC-3′) (Fig. 1a). Amplifications were performed using Taq DNA Polymerase (Qiagen) according to the manufacturer’s protocol with slight modifications: Betaine (final concentration 0.5 M) was used in place of the manufacturers proprietary Q-solution. Reactions were grouped according to product length and performed using the same PCR thermocycler and 384-well plates described above. An initial 5 min denaturation step at 95 °C was followed by 35 cycles of amplification comprising a denaturation step at 94 °C for 30 s, an annealing step at 52 °C for 30 s and an elongation step at 72 °C between 1:30 and 4:00 min. A final elongation step lasting 10 min was performed to ensure complete template generation.

PCR products were visualized on a 1.3% agarose gel and the fragment size was manually verified for all genes, all of which showed the expected fragment length. The products of the second PCR were directly (without purification) used as the expression construct for the following cell-free expression (Fig. 1a).

### Generation of Ni-NTA slides

All microarrays were generated using nickel nitrilotriacetic acid (Ni-NTA) slides as solid support. Epoxysilane coated slides (Schott) were incubated in a solution of 0.63 M NTA and 2.38 M sodium bicarbonate overnight, washed with water twice, air-dried and placed into a 1% nickel sulfate solution for six hours. After another washing step, the slides were incubated in 0.2 M acetic acid, 0.2 M CaCl_2_ and 0.1% Tween20 for 30 minutes, washed, air dried and stored at 4 °C. All steps were performed in a dust-free environment in a sterile hood.

### Generation of protein microarrays

High-density protein microarrays expressing *in situ* the entire Ct proteome were generated using Multiple Spotting Technique^23^. During the first spotting step, 0.6 nl of the product of the second PCR were transferred onto Ni-NTA slides using a Nanoplotter 2 (GeSIM). Subsequently, 2.4 nl of the S30 T7 High-Yield Protein Expression Kit (Promega) were transferred directly on top of the expression construct spots. The slides were then incubated in microarray hybridization cassettes (Arrayit Corporation) at 37 °C for 1 hour and at 30 °C overnight in a humidified environment. The expressed proteins were immobilized to the nickel surface of the microarray slide. The C-terminal V5 sequence allowed the detection of full length expressed proteins. Arrays were stored at −20 °C for up to 3 months without loss of reactivity.

### Determination of on-chip expression

Success of protein expression on the microarray was determined by incubation with fluorescence-conjugated antibodies directed against the N- and C-terminal fusion tags. Slides mounted in single chamber frames were blocked with 2 ml SuperBlock blocking buffer (Thermo Scientific) in ProPlate Slide Modules (Grace Bio-Labs) on an orbital shaker at room temperature for 45 min. Subsequently, they were washed twice for 5 min with 2 ml phosphate-buffered saline containing 0.05% Tween20 (PBST) on a shaker. Fluorescence-conjugated antibodies (Anti-6xHis, DyLight 650 (Abcam) and Anti-V5, Cy3 conjugate (Sigma-Aldrich)) were diluted 1:1000 in blocking buffer. One ml of antibody-dilution was pipetted onto the slide and incubated at room temperature for 1 h on an orbital shaker. Thereafter, the slides were removed from the modules, washed three times with PBST for 10 min, rinsed in sterile-filtered water and air-dried in a ventilated oven at 30 °C. The slides were scanned using a Power Scanner (Tecan) at 532 nm and 635 nm excitation wavelengths, respectively, and analyzed using the microarray acquisition and analysis software GenePix Pro 6.0 (Molecular Devices). Signal intensities were measured as median fluorescence intensity (MFI) signal of all pixels measured for one protein. Signal intensity was considered to be representative of the amount of expressed protein on the slide. Final MFI values were calculated by subtracting the background signal surrounding each individual spot, and the signal of the first negative control (NC1). For NC1, both successive PCRs were performed without DNA template and the product of the second PCR was spotted as template for on-chip protein expression. A protein was considered to be expressed if its signal intensity generated by the labeled antibodies to either the 6xHis or the V5 tag was higher than the mean plus five standard deviations of 20 NC1 replicates.

### Proteome Immunoassay

Protein microarrays displaying 898 potential antigens of Ct (including one Cp antigen) were blocked and washed as described above. Serum samples (in pools or individually) were diluted 1:33 in blocking buffer containing 1 µg/µl *E. coli* wildtype lysate in order to block serum antibodies directed against *E. coli* proteins. Proteins of the *E. coli* based expression mixture for cell-free protein expression might have bound to the microarray slide and would otherwise be able to capture *E. coli* antibodies in applied serum samples. After serum incubation on microarrays at room temperature for 1 h on an orbital shaker, the microarrays were washed twice with PBST as described above and incubated with a 1:350 dilution of a secondary antibody (Alexa Fluor 647-conjugated goat anti-human IgA, IgG, IgM; Jackson Immuno Research) for 1 h on a shaker. The microarrays were again washed and scanned at an excitation wavelength of 635 nm. The signal intensity obtained for a given antigen was considered proportional to the amount of primary antibody bound on the microarray. Final MFIs were generated as described for determination of on-chip expression. Antigen-specific signals with pooled or single serum samples were considered seropositive if they exceeded the mean plus 5 standard deviations of 20 NC1 replicates.

### Human Sera

Sera were part of a Mongolian population-based cross-sectional HPV prevalence study comprising sera of 1002 women (median age 36 years, range 15 to 59)^27^, and an accompanying series of 96 histology-confirmed CxCa patients (median age 49; range 30–77)^28,35^. The study was approved by the ethical review committees of the International Agency for Research on Cancer (IARC) and the Ministry of Health in Mongolia, and all study participants provided informed consent. We hereby confirm that all experiments were performed in accordance with relevant guidelines and regulations. In total, for 985 of the 1002 women, epidemiological questionnaire data, serum and cervical liquid-based cytology samples were available.

Ct serostatus was determined by multiplex serology^26^ using the five immunogenic Ct proteins CT_110 (HSP60, GroEL)^36^, CT_681 (major outer membrane protein, MOMP)^36^, CT_456 (translocated actin-recruiting phosphoprotein, TARP)^37^, CT_713 (outer membrane protein, PORB)^38^, and plasmid-encoded protein pGP3^13^ as antigens. We analyzed sera from women with defined Ct-DNA status: Ct-DNA + (n = 85) or Ct-DNA- (n = 29, < 22 years, ≤ 1 lifetime sexual partner). Using Ct-DNA status in the two reference groups as gold-standard, maximum values for sensitivity and specificity were achieved when Ct seropositivity was defined as antibody response to 2 or more individual proteins or to MOMPmax >1000 MFI alone (sensitivity 83% and specificity 87%, respectively) (Hulstein *et al*., submitted; Trabert *et al*., submitted). Women who were Ct seronegative and Ct DNA negative were designated Ct-uninfected controls. Women who were Ct DNA and/or seropositive were designated Ct-infected.

CxCa patients were recruited based on clinical and local histopathological diagnosis in Ulaanbaatar and were further characterized in detail for HPV antibodies and HPV DNA prevalence resulting in 96 confirmed CxCa cases (median age 49; range 30–77)^28^.

A case-control study was designed based on the 96 CxCa cases and 520 controls (>30 years and showing no histological abnormality) of the Mongolian population-based cross-sectional HPV prevalence study^27^.

### Multiplex serology

Multiplex serology is a bead based suspension array technology and was performed as described previously by Waterboer *et al*. (2005). Briefly, selected Ct antigens were expressed as recombinant glutathione S-transferase (GST) fusion proteins and loaded on glutathione-casein coupled spectrally distinct fluorescence-labeled polystyrene beads (SeroMap; Luminex). Antigen-loaded beads were mixed and incubated with sera using a final serum dilution of 1:100. Serum antibodies bound to antigen-loaded beads were quantified using a biotinylated goat anti-human IgG, IgM, IgA secondary antibody (Jackson Immuno Research). Fluorescent signals were generated by adding the reporter conjugate streptavidin R-phycoerythrin and measured using a Luminex 200 analyzer. Final MFI values were calculated by subtracting the MFI value of GST only (i.e., without bacterial protein fusion component) and individual bead background values^26^.

### Statistical methods

Reproducibility and stability of Ct whole-proteome microarrays were investigated by linear regression analysis and Pearson’s correlation coefficient (r). Receiver Operating Characteristic (ROC) analysis was performed to determine cut-offs for newly identified antigens maximizing sensitivity and specificity. Differences in continuous MFI values between comparison groups were assessed by Mann-Whitney tests. Odds ratios (OR) and 95% confidence intervals (CI) were calculated using unconditional logistic regression adjusting for age, any high-risk HPV L1 serology, herpes simplex virus 1 (HSV1) serology, education, number of deliveries and number of lifetime sexual partners.

All analyses were performed with Microsoft Excel, GraphPad Prism, and SAS Version 9.4; p-values ≤0.05 were considered statistically significant.

### Data Availability

The datasets generated during and/or analyzed during the current study are available from the corresponding author on reasonable request.

## Electronic supplementary material

Supplementary Information
