# Supplementary material for: Immunoprofiling of Chlamydia trachomatis using whole-proteome microarrays generated by on-chip in situ expression
Source: Sci Rep. 2018 May 14;8:7503. doi: 10.1038/s41598-018-25918-3 (PMC5951824; doi:10.1038/s41598-018-25918-3)
Supplement: Supplementary file 1 — Supplementary Information [file 41598_2018_25918_MOESM1_ESM.pdf]

Immunoprofiling of *Chlamydia trachomatis* using whole-proteome microarrays generated by on-chip *in situ* expression

Katrin Hufnagel*[1], Smiths Lueong[1], Martina Willhauck-Fleckenstein[1], Agnes Hotz-Wagenblatt[2], Beiping Miao[3], Andrea Bauer[3], Angelika Michel[1], Julia Butt[1], Michael Pawlita[1], Jörg D. Hoheisel[3], Tim Waterboer[1]

Supplementary tables and figures

Supplementary table S1: List of all *Chlamydia trachomatis* proteins covered by whole proteome arrays. Expression signals for N- and C-terminal expression were measured in median fluorescence intensities (MFI). Cut-offs for N- and C-terminal expression are 33.11 and 82.41 MFI, respectively. Antigens, selected to be analyzed using smaller microarrays and single serum samples were highlighted in gray.

***Chlamydia trachomatis* D/UW-3/CX chromosome: 887 proteins**					**Expression Signal [MFI]**	
**Synonym**	**Gene**	**Strand**	**Product**	**Length [aa]**	**N-Terminal**	**C-Terminal**
CT_001	-	-	hypothetical protein	90	2025	12492
CT_002	gatC	+	glutamyl-tRNA(Gln) amidotransferase subunit C	100	1531	7825
CT_003	gatA	+	glutamyl-tRNA(Gln) amidotransferase subunit A	491	174	18
CT_004	gatB	+	aspartyl/glutamyl-tRNA(Asn/Gln) amidotransferase subunit B	488	192	487
CT_005	-	-	hypothetical protein	363	11901	798
CT_006	-	-	hypothetical protein	189	12714	9136
CT_007	-	+	hypothetical protein	316	996	4002
CT_008	rnhB_1	-	ribonuclease HII	300	138	1133
CT_009	yfgA	+	HTH transcriptional regulator	143	820	8481
CT_010	htrB	-	acyltransferase	455	13	370
CT_011	-	-	hypothetical protein	418	2737	9062
CT_012	ybbP	-	hypothetical protein	264	17548	1183
CT_013	cydA	+	cytochrome oxidase subunit I	446	23	80
CT_014	cydB	+	cytochrome oxidase subunit II	353	19364	1246
CT_015	phoH	-	ATPase	434	3231	4326
CT_016	-	+	hypothetical protein	242	5507	5476
CT_017	-	+	hypothetical protein	433	330	2072

CT_018	-	-	hypothetical protein	157	1070	6470
CT_019	ileS	+	isoleucine--tRNA ligase	1036	316	281
CT_020	lepB	-	signal peptidase I	628	1441	344
CT_021	-	-	hypothetical protein	247	3425	29587
CT_022	rl31	+	50S ribosomal protein L31	108	247	3121
CT_023	prfA	+	peptide chain release factor	359	3263	619
CT_024	-	+	release factor glutamine methyltransferase	290	193	1753
CT_025	ffh	+	signal recognition particle GTPase	448	32	65
CT_026	rs16	+	30S ribosomal protein S16	116	1155	32218
CT_027	trmD	+	tRNA (guanine N-1) methyltransferase	352	1192	4273
CT_028	rl19	+	50S ribosomal protein L19	121	427	14239
CT_029	rnhB_2	+	ribonuclease HII	217	161	4331
CT_030	gmk	+	guanylate kinase	205	3049	8930
CT_031	-	+	hypothetical protein	100	31	1921
CT_032	metG	+	methionine--tRNA ligase	550	24	1402
CT_033	recD_1	-	exodeoxyribonuclease V subunit alpha	746	116	876
CT_034	ytfF	-	cationic amino acid transporter	341	7899	1196
CT_035	-	+	hypothetical protein	257	551	1206
CT_036	-	-	hypothetical protein	403	4959	803
CT_037	-	-	hypothetical protein	118	7889	4375
CT_038	-	-	hypothetical protein	116	4465	4202
CT_039	dcd	-	deoxycytidine triphosphate deaminase	190	4536	14148
CT_039.1	-	+	hypothetical protein	50	1546	9534
CT_040	ruvB	+	Holliday junction ATP-dependent DNA helicase RuvB	334	153	17
CT_041	-	+	hypothetical protein	270	415	2087
CT_042	glgX	-	glycogen hydrolase	666	624	530
CT_043	-	-	hypothetical protein	167	2341	9545
CT_044	ssb	+	single-stranded DNA-binding protein	157	779	5518
CT_045	pepA	+	cytosol aminopeptidase	499	1407	1755
CT_046	hctB	+	histone-like protein	203	205	3307
CT_047	-	+	hypothetical protein	314	211	5

CT_048	yraL	+	SAM-dependent methyltransferase	237	3964	1770
CT_049	-	+	hypothetical protein	490	3058	622
CT_050	-	-	hypothetical protein	536	92	18
CT_051	-	-	hypothetical protein	520	80	42
CT_052	hemN_1	-	coproporphyrinogen III oxidase	378	112	312
CT_053	-	-	hypothetical protein	148	123	6189
CT_054	sucA	+	oxoglutarate dehydrogenase	905	7127	637
CT_055	sucB_1	+	dihydrolipoamide succinyltransferase	365	12818	1208
CT_056	-	-	hypothetical protein	243	628	10756
CT_057	gcpE	-	4-hydroxy-3-methylbut-2-en-1-yl diphosphate synthase	602	335	778
CT_058	-	-	hypothetical protein	367	579	1085
CT_059	fer	-	ferredoxin	91	90	4403
CT_060	-	+	type III secretion system protein	605	3325	546
CT_061	-	+	RNA polymerase sigma factor sigma-28	253	1174	4245
CT_062	tyrS	+	tyrosine--tRNA ligase	412	1208	846
CT_063	gnd	+	6-phosphogluconate dehydrogenase	480	23	584
CT_064	lepA	-	elongation factor EF-4	602	32	86
CT_065	-	-	ADP/ATP translocase	528	60010	2446
CT_066	-	-	hypothetical protein	158	1749	18851
CT_067	ytgA	+	metal ABC transporter substrate-binding lipoprotein	326	1133	1426
CT_068	ytgB_1	+	metal ABC transporter ATP-binding protein	259	3496	4291
CT_069	ytgC	+	metal ABC transporter permease	451	466	201
CT_070	ytgD	+	metal ABC transporter permease	318	306	1271
CT_071	yaeM	+	1-deoxy-D-xylulose 5-phosphate reductoisomerase	379	338	2850
CT_072	yaeL	+	zinc metalloprotease	619	141	11
CT_073	-	-	outer membrane protein	325	728	5022
CT_074	recF	-	DNA replication and repair protein RecF	365	611	2542
CT_075	dnaN	-	DNA polymerase III subunit beta	416	16	84
CT_076	smpB	+	SsrA-binding protein	151	14754	8104
CT_077	-	-	thiamine biosynthesis lipoprotein ApbE	316	1594	2907

CT_078	folD	-	bifunctional methylenetetrahydrofolate dehydrogenase/ methenyltetrahydrofolate cyclohydrolase	287	59	680
CT_079	-	-	hypothetical protein	147	11286	18015
CT_080	ltuB	+	late transcription unit protein B	97	507	15283
CT_081	-	+	hypothetical protein	98	20629	1554
CT_082	-	+	hypothetical protein	560	27	401
CT_083	-	+	hypothetical protein	160	823	4032
CT_084	-	-	phospholipase D superfamily protein	361	546	1676
CT_085	-	-	hypothetical protein	579	32	1080
CT_086	rl28	-	50S ribosomal protein L28	89	112	1321
CT_087	malQ	-	4-alpha glucanotransferase	527	2005	612
CT_088	sycE	-	secretion chaperone	146	21492	16184
CT_089	lcrE	-	low calcium response protein E	421	17	78
CT_090	lcrD	-	low calcium response protein D	708	80	186
CT_091	-	-	type III secretion system protein	360	6499	5716
CT_092	ychF	+	GTPase	366	826	1172
CT_093	ribF	-	riboflavin kinase/FAD synthase	301	473	3220
CT_094	truB	-	tRNA pseudouridine synthase B	241	1753	6065
CT_095	rbfA	-	ribosome binding factor A	123	165	2358
CT_096	infB	-	translation initiation factor IF-2	892	546	1422
CT_097	nusA	-	transcription antitermination factor	434	6	34
CT_098	rs1	-	30S ribosomal protein S1	569	2432	1240
CT_099	trxB	+	thioredoxin reductase	351	2637	1413
CT_100	acpS	+	holo [acyl-carrier protein] synthase	119	1129	1037
CT_101	-	-	hypothetical protein	153	24314	8022
CT_102	-	+	hypothetical protein	153	2748	8656
CT_103	-	+	HAD superfamily hydrolase/phosphatase	298	2320	3122
CT_104	fabI	-	enoyl-acyl-carrier protein reductase	298	2538	2274
CT_105	-	+	hypothetical protein	656	112	954
CT_106	yceC	-	pseudouridine synthase	303	400	3502

CT_107	mutY	+	A/G-specific adenine glycosylase	369	86	235
CT_108	-	+	hypothetical protein	251	8985	7581
CT_109	-	-	hypothetical protein	262	451	709
CT_110	groEL_1	-	chaperonin GroEL	544	134	822
CT_111	groES	-	co-chaperonin GroES	102	141	7465
CT_112	pepF	-	oligoendopeptidase	608	68	139
CT_113	clpB	+	chaperone protein ClpB	867	811	954
CT_114	-	+	hypothetical protein	486	43	1228
CT_115	-	+	inclusion membrane protein D	141	9149	8671
CT_116	-	+	inclusion membrane protein E	132	1032	5050
CT_117	-	+	inclusion membrane protein F	104	5474	12724
CT_118	-	+	inclusion membrane protein G	167	1869	1467
CT_119	incA	-	inclusion membrane protein A	273	12625	4849
CT_120	-	-	hypothetical protein	80	232	5072
CT_121	araD	+	ribulose-phosphate 3-epimerase	233	889	4448
CT_122	efp_1	+	elongation factor P	185	3442	1363
CT_123	accB	+	acetyl-CoA carboxylase biotin carboxyl carrier protein	164	2775	15644
CT_124	accC	+	biotin carboxylase	457	1854	1003
CT_125	rl13	+	50S ribosomal protein L13	150	264	22751
CT_126	rs9	+	30S ribosomal protein S9	129	989	12239
CT_127	ydhO	-	polysaccharide hydrolase-invasin repeat family protein	283	338	806
CT_128	adk	-	adenylate kinase	245	283	1764
CT_129	glnP	+	ABC amino acid transporter permease	214	12284	6771
CT_130	glnQ	+	ABC amino acid transporter ATPase	233	1796	11714
CT_131	-	-	transmembrane protein	1138	14	77
CT_132	-	-	hypothetical protein	425	4489	1521
CT_133	-	-	rRNA methylase	267	88	3389
CT_134	-	+	hypothetical protein	137	3422	2074
CT_135	-	+	hypothetical protein	360	774	183
CT_136	-	+	lysophospholipase esterase	239	572	2144

CT_137	ywlC	+	SuA5 family protein	281	612	1597
CT_138	-	+	dipeptidase	315	18	160
CT_139	oppA_1	-	oligopeptide-binding protein	426	444	1562
CT_140	-	+	hypothetical protein	228	37760	8441
CT_141	secA_1	+	protein translocase SecA	148	72	15150
CT_142	-	+	hypothetical protein	285	15820	6169
CT_143	-	+	hypothetical protein	280	1057	2265
CT_144	-	+	hypothetical protein	285	7578	5999
CT_145	-	+	serine/threonine protein kinase	614	22470	1082
CT_146	dnlJ	+	DNA ligase	663	25	374
CT_147	-	+	hypothetical protein	1449	98	288
CT_148	mhpA	-	monooxygenase	507	340	3087
CT_149	-	-	hydrolase	315	1603	1158
CT_150	rl33	+	50S ribosomal protein L33	52	2835	12711
CT_151	-	+	hypothetical protein	503	2341	1701
CT_152	ycfV	+	ABC transporter ATPase	225	2964	10223
CT_153	-	-	hypothetical protein	810	15	93
CT_154	-	-	phospholipase D endonuclease family protein	383	1998	2748
CT_155	-	-	phospholipase D endonuclease family protein	313	43	14
CT_156	-	-	hypothetical protein	113	305	1157
CT_157	-	-	phospholipase D endonuclease family protein	404	37	75
CT_158	-	-	phospholipase D endonuclease family protein	238	3638	7742
CT_159	-	-	hypothetical protein	310	817	884
CT_160	-	-	hypothetical protein	167	2342	7058
CT_161	-	-	hypothetical protein	246	834	5613
CT_162	-	-	hypothetical protein	79	614	4693
CT_163	-	+	hypothetical protein	548	434	180
CT_164	-	+	hypothetical protein	86	10579	3199
CT_165	-	+	hypothetical protein	148	704	1978
CT_166	-	+	hypothetical protein	639	12	87
CT_167	-	+	hypothetical protein	443	1051	952

CT_168	-	+	hypothetical protein	100	4583	21991
CT_169	trpR	+	Trp operon repressor	94	2412	9718
CT_170	trpB	+	tryptophan synthase subunit beta	392	288	1313
CT_171	trpA	+	tryptophan synthase subunit alpha	253	720	5983
CT_172	-	-	hypothetical protein	163	9525	3591
CT_172.1	-	-	hypothetical protein	56	335	5051
CT_173	-	-	hypothetical protein	90	819	6556
CT_174	-	-	hypothetical protein	151	1225	12343
CT_175	oppA_2	+	oligonucleotide ABC transporter substrate-binding protein	529	125	323
CT_176	dsbB	-	disulfide formation protein	135	5867	5137
CT_177	dsbG	-	disulfide bond chaperone	238	7895	15448
CT_178	-	+	hypothetical protein	404	138	81
CT_179	-	-	hypothetical protein	170	43	1741
CT_180	tauB	-	iron/nitrate ABC transporter ATPase	230	2403	2934
CT_181	-	-	hypothetical protein	236	1321	7949
CT_182	kdsB	+	3-deoxy-manno-octulosonate cytidylyltransferase	254	1473	4942
CT_183	pyrG	+	CTP synthetase	539	12	359
CT_184	-	+	Holliday junction resolvase	148	7336	2313
CT_185	zwf	+	glucose-6-phosphate 1-dehydrogenase	439	848	1356
CT_186	devB	+	6-phosphogluconolactonase	256	1799	1747
CT_187	dnaX_1	-	DNA polymerase III subunit gamma and tau	290	446	1672
CT_188	tdk	-	thymidylate kinase	203	532	10739
CT_189	gyrA_1	-	DNA gyrase subunit A	836	12939	472
CT_190	gyrB_1	-	DNA gyrase subunit B	804	13	33
CT_191	-	-	hypothetical protein	116	1290	1756
CT_192	-	-	hypothetical protein	257	2370	10879
CT_193	tgt	-	queuine tRNA ribosyltransferase	372	194	1911
CT_194	mgtE	-	Mg++ transporter	470	1789	1429
CT_195	-	-	hypothetical protein	363	250	581
CT_196	-	+	hypothetical protein	106	27367	11553

CT_197	-	+	metal dependent protease	338	97	362
CT_198	oppA_3	+	oligopeptide ABC transporter substrate-binding protein	518	26	119
CT_199	oppB_1	+	oligopeptide ABC transporter permease	313	18249	2300
CT_200	oppC_1	+	oligopeptide ABC transporter permease	281	30393	1935
CT_201	oppD	+	oligopeptide ABC transporter ATPase	277	24	169
CT_202	oppF	+	oligopeptide ABC transporter ATPase	247	362	1382
CT_203	-	+	hypothetical protein	251	1790	52908
CT_204	ybhI	+	dicarboxylate translocator	471	1054	498
CT_205	pfkA_1	+	fructose-6-phosphate phosphotransferase	553	47	757
CT_206	-	+	acyltransferase family protein	282	6799	9658
CT_207	pfkA_2	+	fructose-6-phosphate phosphotransferase	548	36	273
CT_208	gseA	-	3-deoxy-D-manno-octulosonic acid transferase	431	7450	767
CT_209	leuS	-	leucine--tRNA ligase	819	72	613
CT_210	hemL	+	glutamate-1-semialdehyde-2,1-aminomutase	422	408	1148
CT_211	-	-	hypothetical protein	189	49	2881
CT_212	-	-	hypothetical protein	148	45909	4337
CT_213	rpiA	-	ribose-5-phosphate isomerase A	242	784	4968
CT_214	-	-	hypothetical protein	547	37	6
CT_215	dhnA	+	1,6-fructose biphosphate aldolase	348	33	478
CT_216	xasA	+	amino acid transporter	466	5957	1200
CT_217	ydaO	+	PP-loop superfamily ATPase	234	1645	12707
CT_218	surE	+	5'-nucleotidase SurE	283	2881	1160
CT_219	ubiA	+	4-hydroxybenzoate octaphenyltransferase	302	133	17
CT_220	ubiX	+	aromatic acid decarboxylase	192	341	9421
CT_221	yqfU	-	hypothetical protein	298	33011	2836
CT_223	-	-	inclusion membrane protein	270	9842	4546
CT_224	-	-	hypothetical protein	147	19380	11275
CT_225	-	-	hypothetical protein	122	6323	3773
CT_226	-	-	hypothetical protein	176	2276	6146
CT_227	-	-	hypothetical protein	133	722	3110

CT_228	-	-	hypothetical protein	196	7677	12399
CT_229	-	-	hypothetical protein	215	9498	5139
CT_230	-	+	neutral amino acid transporter	415	18612	4633
CT_231	-	+	Ssodium-dependent amino acid transporter	490	8771	1146
CT_232	incB	+	inclusion membrane protein B	115	19440	16832
CT_233	incC	+	inclusion membrane protein C	178	7729	15644
CT_234	-	+	hypothetical protein	928	204	171
CT_235	-	+	cAMP-dependent protein kinase regulatory subunit	137	10794	13713
CT_236	acpP	-	acyl carrier protein	77	2106	3960
CT_237	fabG	-	oxoacy-ACP reductase	248	819	5758
CT_238	fabD	-	malonyl CoA-ACP transacylase	308	616	611
CT_239	fabH	-	oxoacyl-ACP synthase III	327	914	3462
CT_240	recR	+	recombination protein RecR	200	2124	1656
CT_241	yaeT	+	outer membrane protein	792	647	1874
CT_242	-	+	OmpH-Like outer membrane protein	173	3157	5967
CT_243	lpxD	+	UDP-3-O-acylglucosamine N-acyltransferase	354	1508	3597
CT_244	-	-	hypothetical protein	398	1295	1019
CT_245	pdhA	+	pyruvate dehydrogenase subunit alpha	340	1712	1337
CT_246	pdhB	+	pyruvate dehydrogenase subunit beta	328	1162	6645
CT_247	pdhC	+	dihydrolipoamide acetyltransferase	429	3371	934
CT_248	glgP	-	glycogen phosphorylase	814	16	52
CT_249	-	+	hypothetical protein	116	8759	24699
CT_250	dnaA_1	-	chromosomal replication initiator protein DnaA	456	10102	1834
CT_251	-	-	membrane protein insertase	787	1438	556
CT_252	lgt	-	prolipoprotein diacylglycerol transferase	272	6806	1706
CT_253	-	+	hypothetical protein	215	12297	3960
CT_254	-	+	hypothetical protein	256	3034	3742
CT_255	-	-	hypothetical protein	127	4029	9331
CT_256	-	+	hypothetical protein	414	347	758
CT_257	-	+	hypothetical protein	404	305	136
CT_258	yhfO	-	NifS family protein	374	61	293

CT_259	-	-	PP2C phosphatase family protein	248	1856	4673
CT_260	-	+	hypothetical protein	163	2558	13739
CT_261	dnaQ_1	+	DNA polymerase III subunit epsilon	232	881	2471
CT_262	-	+	hypothetical protein	256	454	2257
CT_263	-	+	hypothetical protein	196	6327	5713
CT_264	msbA	-	ABC transporter ATP binding protein/permease	646	9242	1442
CT_265	accA	-	acetyl-coenzyme A carboxylase carboxyl transferase subunit alpha	324	2317	2398
CT_266	-	-	hypothetical protein	393	7272	1408
CT_267	ihfA	-	DNA-binding protein HU	100	803	12454
CT_268	amiA	-	N-acetylmuramoyl alanine amidase	259	3510	6215
CT_269	murE	-	UDP-N-acetylmuramoyl-L-alanyl-D-glutamate-2,6-diaminopimelate ligase	483	207	657
CT_270	pbp3	-	transglycolase/transpeptidase	647	4271	269
CT_271	-	-	hypothetical protein	95	2531	12601
CT_272	-	-	ribosomal RNA small subunit methyltransferase H	300	430	1226
CT_273	-	+	hypothetical protein	188	987	2180
CT_274	-	+	hypothetical protein	139	10109	5579
CT_275	dnaA_2	+	chromosomal replication initiator protein DnaA	455	48	331
CT_276	-	+	hypothetical protein	194	193	7242
CT_277	-	-	hypothetical protein	219	2574	2945
CT_278	nqr2	+	NADH-quinone reductase subunit B	503	826	876
CT_279	nqr3	+	NADH-quinone reductase subunit C	316	25	198
CT_280	nqr4	+	NADH-quinone reductase subunit D	213	37466	5932
CT_281	nqr5	+	NADH-quinone reductase subunit E	244	13077	7548
CT_282	gcsH	-	glycine cleavage system protein H	117	2383	10885
CT_283	-	-	hypothetical protein	698	467	393
CT_284	-	-	phospholipase D superfamily protein	474	2087	584
CT_285	lplA_1	-	lipoate protein ligase	239	313	3657
CT_286	clpC	+	ATP-dependent Clp protease ATP-binding subunit ClpC	854	81	151

CT_287	-	-	tRNA-specific 2-thiouridylase MnmA	358	134	842
CT_288	-	+	hypothetical protein	563	40	32
CT_289	-	-	hypothetical protein	379	235	837
CT_290	ptsN_1	-	PTS system transporter subunit IIA	225	2987	4782
CT_291	ptsN_2	-	PTS system transporter subunit IIA	158	2391	7065
CT_292	dut	-	deoxyuridine 5'-triphosphate nucleotidohydrolase	145	67	8911
CT_293	accD	-	acetyl-coenzyme A carboxylase carboxyl transferase subunit beta	308	68	1394
CT_294	sodM	-	superoxide dismutase	206	3011	8921
CT_295	mrsA_1	-	phosphomannomutase	593	585	141
CT_296	-	-	hypothetical protein	155	3659	4612
CT_297	rnc	+	ribonuclease III	231	5679	11919
CT_298	radA	+	DNA repair protein RadA	454	538	552
CT_299	hemC	+	porphobilinogen deaminase	241	102	1728
CT_300	-	+	hypothetical protein	115	5626	9640
CT_301	pknD	-	serine/threonine-protein kinase	934	79	69
CT_302	valS	-	valine--tRNA ligase	939	19	63
CT_303	-	-	hypothetical protein	171	6249	6029
CT_304	atpK	-	V-type ATP synthase subunit K	141	4668	10317
CT_305	atpI	-	V-type ATP synthase subunit I	649	41	95
CT_306	atpD	-	V-type ATP synthase subunit D	203	834	7646
CT_307	atpB	-	V-type ATP synthase subunit B	438	135	425
CT_308	atpA	-	V-type ATP synthase subunit A	591	17	83
CT_309	-	-	hypothetical protein	266	170	2034
CT_310	atpE	-	V-type ATP synthase subunit E	208	1059	7101
CT_311	-	+	hypothetical protein	236	2001	13766
CT_312	-	-	ferredoxin	123	606	3573
CT_313	tal	-	transaldolase	327	189	901
CT_314	rpoC	-	DNA-directed RNA polymerase subunit beta'	1396	212	51
CT_315	rpoB	-	DNA-directed RNA polymerase subunit beta	1252	14	24
CT_316	rl7	-	50S ribosomal protein L7/L12	130	162	4349

CT_317	rl10	-	50S ribosomal protein L10	172	3236	2001
CT_318	rl1	-	50S ribosomal protein L1	232	607	8285
CT_319	rl11	-	50S ribosomal protein L11	141	1063	3386
CT_320	nusG	-	transcription termination/antitermination protein NusG	182	712	5844
CT_321	secE	-	protein translocase subunit SecE	82	12278	6989
CT_322	tufA	-	elongation factor Tu	394	1135	816
CT_323	infA	-	translation initiation factor IF-1	73	369	49477
CT_324	-	+	hypothetical protein	303	8451	6586
CT_325	-	+	hypothetical protein	148	13830	3201
CT_326	-	-	hypothetical protein	563	20	287
CT_326.1	-	-	hypothetical protein	62	265	1335
CT_326.2	-	-	hypothetical protein	60	111	7732
CT_327	trpC	-	N-(5'-phosphoribosyl)anthranilate isomerase	208	2419	6743
CT_328	tpiS	+	triosephosphate isomerase	274	414	2885
CT_329	xseA	+	exodeoxyribonuclease VII large subunit	516	756	605
CT_330	-	+	hypothetical protein	90	356	4522
CT_331	dxs	+	1-deoxy-D-xylulose-5-phosphate synthase	640	33	225
CT_332	pykF	+	pyruvate kinase	485	21	225
CT_333	uvrA	+	excinuclease ABC subunit A	1786	54	149
CT_334	dnaX_2	+	DNA polymerase III subunit gamma and tau	466	180	2019
CT_335	-	+	hypothetical protein	96	55	15486
CT_336	ptsI	-	PTS system PEP phosphotransferase	571	1694	390
CT_337	ptsH	-	PTS system phosphocarrier protein Hpr	109	3227	4567
CT_338	-	-	hypothetical protein	153	10826	8843
CT_339	-	+	hypothetical protein	509	180	20
CT_340	pdhA/B	-	oxoisovalerate dehydrogenase subunits alpha/beta	678	1007	821
CT_341	dnaJ	-	heat shock protein DnaJ	392	160	2668
CT_342	rs21	-	30S ribosomal protein S21	58	30	1000
CT_343	-	-	O-sialoglycoprotein endopeptidase family protein	210	1288	29309
CT_344	lon	+	Lon ATP-dependent protease	819	864	358
CT_345	-	+	hypothetical protein	121	7113	11689

CT_346	elaC	+	ribonuclease Z	304	73	514
CT_347	xerC	+	tyrosine recombinase XerC	315	105	1157
CT_348	yjjK	+	ABC transporter ATPase	528	13	47
CT_349	maf	+	Maf protein	196	1945	7427
CT_350	-	+	hypothetical protein	566	1501	874
CT_351	-	+	hypothetical protein	697	38	142
CT_352	-	+	hypothetical protein	101	94	1815
CT_353	def	-	peptide deformylase	181	1983	3223
CT_354	kgsA	-	rRNA small subunit methyltransferase A	277	61	222
CT_355	-	-	hypothetical protein	353	21884	4055
CT_356	yyaL	-	thioredoxin domain-containing protein	704	129	106
CT_357R	-	-	hypothetical protein	95	3965	11830
CT_357	-	+	hypothetical protein	110	4438	3516
CT_358	-	+	hypothetical protein	178	544	1364
CT_359	-	-	hypothetical protein	196	23229	4615
CT_360	-	-	hypothetical protein	208	4027	6676
CT_361	dapA	-	4-hydroxy-tetrahydrodipicolinate synthase	286	2845	1189
CT_362	lysC	-	aspartokinase	431	31	350
CT_363	asd	-	aspartate semialdehyde dehydrogenase	334	54	219
CT_364	dapB	-	4-hydroxy-tetrahydrodipicolinate reductase	253	34	688
CT_365	-	-	hypothetical protein	575	14	24
CT_366	aroA	+	phosphoshikimate 1-carboxyl vinyltransferase	440	42	400
CT_367	aroL	+	shikimate kinase	184	709	2859
CT_368	aroC	+	chorismate synthase	357	90	1909
CT_369	aroB	+	dehyroquinate synthase	373	2143	1158
CT_370	aroE	+	bifunctional 3-dehydroquinate dehydratase/ shikimate dehydrogenase	478	114	144
CT_371	-	+	outer membrane protein	261	175	1251
CT_372	-	+	porin	442	142	1696
CT_373	-	+	pyruvoyl-dependent arginine decarboxylase	195	2094	2724
CT_374	arcD	+	arginine/agmatine antiporter	483	924	521

CT_375	-	+	D-amino acid dehydrogenase	352	1185	4209
CT_376	mdhC	-	malate dehydrogenase	326	27	177
CT_377	ltuA	-	late transcription unit protein A	46	5435	65411
CT_378	pgi	-	glucose-6-phosphate isomerase	525	1177	870
CT_379	hflX	-	GTPase HflX	447	476	2817
CT_380	phnP	-	metal dependent hydrolase	266	3317	2664
CT_381	artJ	-	arginine ABC transporter substrate-binding protein ArtJ	257	2495	4584
CT_382	aroG	-	phospho-2-dehydro-3-deoxyheptonate aldolase	278	7179	5192
CT_382.1	-	-	hypothetical protein	63	1527	16272
CT_383	-	+	hypothetical protein	243	562	3944
CT_384	-	-	hypothetical protein	539	170	598
CT_385	ycfF	-	Hit family hydrolase	111	4648	13900
CT_386	-	-	metal dependent hydrolase	289	3577	4616
CT_387	-	+	hypothetical protein	691	23	330
CT_388	-	-	hypothetical protein	115	4924	12523
CT_389	-	+	hypothetical protein	408	1565	1453
CT_390	aspC	+	LL-diaminopimelate aminotransferase	394	18	135
CT_391	-	+	hypothetical protein	335	1150	843
CT_392	yprS	-	hypothetical protein	377	1993	1954
CT_393	proS	+	proline--tRNA ligase	581	264	498
CT_394	hrcA	+	heat-inducible transcription repressor HrcA	392	4486	7409
CT_395	grpE	+	protein GrpE	190	2824	5355
CT_396	dnaK	+	chaperone protein DnaK	660	14	68
CT_397	vacB	+	ribonuclease R	694	14	65
CT_398	-	+	hypothetical protein	254	1061	2729
CT_399	yrbH	-	GutQ/KpsF family sugar-phosphate isomerase	328	1342	1307
CT_400	sucB_2	-	dihydrolipoamide succinyltransferase	388	1709	2421
CT_401	gltT	-	glutamate symporter	412	338	895
CT_402	lpxK	-	tetraacyldisaccharide 4' kinase	419	1385	1226
CT_403	yjfH	-	SpoU family rRNA methylase	269	1546	3215

CT_404	-	-	SAM dependent methyltransferase	275	115	19
CT_405	ribC	-	riboflavin synthase	199	367	11236
CT_406	nrdR	+	transcriptional repressor NrdR	154	621	5552
CT_407	dksA	+	DnaK suppressor	124	554	944
CT_408	lspA	+	lipoprotein signal peptidase	167	14362	9742
CT_409	-	+	amino acid permease	453	489	1389
CT_410	pcnB_1	+	poly A polymerase	425	872	494
CT_411	lpxB	+	lipid A disaccharide synthase	607	38805	269
CT_412	pmpA	+	outer membrane protein PmpA	975	27	67
CT_413	pmpB	+	outer membrane protein PmpB	1751	166	518
CT_414	pmpC	+	outer membrane protein PmpC	1770	77	1032
CT_415	yebL	+	metal ABC transporter substrate-binding protein	276	7368	1364
CT_416	-	+	metal ABC transporter ATPase	236	1645	3882
CT_417	-	+	metal ABC transporter permease	293	8983	3063
CT_418	-	-	GTPase Obg	335	112	633
CT_419	rl27	-	50S ribosomal protein L27	83	209	7984
CT_420	rl21	-	50S ribosomal protein L21	107	3146	30466
CT_421	-	+	hypothetical protein	233	31304	5044
CT_421.1	-	+	hypothetical protein	53	33	2162
CT_421.2	-	+	hypothetical protein	53	24	3010
CT_422	-	+	endoribonuclease	161	465	3258
CT_423	-	+	CBS domain-containing protein	369	801	5629
CT_424	rsbV_1	+	anti-sigma factor antagonist	116	418	3653
CT_425	-	+	hypothetical protein	621	4606	534
CT_426	-	+	Fe-S oxidoreductase	369	736	1116
CT_427	-	+	hypothetical protein	273	484	1004
CT_428	ubiE	+	demethylmenaquinone methyltransferase	229	3674	2486
CT_429	-	-	hypothetical protein	329	486	541
CT_430	dapF	-	diaminopimelate epimerase	275	264	725
CT_431	clpP_1	-	ATP-dependent Clp protease proteolytic subunit	192	200	3184
CT_432	glyA	-	serine hydroxymethyltransferase	497	1350	694

CT_433	-	+	hypothetical protein	223	544	1378
CT_434	ygbB	+	2-C-methyl-D-erythritol 2,4-cyclodiphosphate synthase	178	358	4986
CT_435	cysJ	-	sulfite reductase	350	1467	1491
CT_436	rs10	-	30S ribosomal protein S10	105	781	13594
CT_437	fusA	-	elongation factor G	694	39602	1713
CT_438	rs7	-	30S ribosomal protein S7	157	64	7414
CT_439	rs12	-	30S ribosomal protein S12	129	3457	24130
CT_440	-	+	hypothetical protein	112	14780	42761
CT_441	tsp	+	tail-specific protease	644	20	62
CT_442	crpA	-	cysteine-rich protein	150	26084	8489
CT_443	omcB	-	cysteine-rich outer membrane protein OmcB	553	224	599
CT_444	omcA	-	cysteine-rich lipoprotein OmcA	88	56	8942
CT_444.1	-	+	hypothetical protein	74	1649	10536
CT_445	gltX	-	glutamate--tRNA ligase	506	1547	1188
CT_446	-	-	hypothetical protein	183	60173	17225
CT_447	recJ	-	ssDNA exonuclease	584	81	708
CT_448	secD/secF	-	bifunctional preprotein translocase subunit SecD/SecF	1400	19	37
CT_449	-	-	hypothetical protein	110	7096	19993
CT_450	-	+	isoprenyl transferase	253	612	10567
CT_451	cdsA	+	phosphatidate cytidylytransferase	305	25973	588
CT_452	cmk	+	cytidylate kinase	216	97	1829
CT_453	plsC	+	glycerol-3-phosphate acyltransferase	216	518	2985
CT_454	argS	+	arginine--tRNA ligase	563	19756	3225
CT_455	murA	-	UDP-N-acetylglucosamine 1-carboxyvinyltransferase	444	3495	3052
CT_456	-	+	translocated actin-recruiting phosphoprotein	1005	212	59
CT_457	-	-	transcriptional regulator	238	1689	3342
CT_458	YhhY	-	acetyltransferase	167	418	9854
CT_459	prfB	-	peptide chain release factor 2	369	58	229
CT_460	-	+	SWIB complex protein	86	2665	7371

CT_461	-	+	metallophosphoesterase	329	208	233
CT_462	-	+	2-C-methyl-D-erythritol 4-phosphate cytidylyltransferase	219	506	3513
CT_463	truA	+	tRNA pseudouridine synthase A	267	596	1289
CT_464	-	-	phosphoglycolate phosphatase	224	2381	3708
CT_465	-	+	hypothetical protein	213	749	4686
CT_466	-	+	hypothetical protein	109	596	2025
CT_467	atoS	+	two component regulatory system sensor histidine kinase	352	2967	1672
CT_468	atoC	+	two component regulatory system response regulator/ATPase	386	40	197
CT_469	-	+	hypothetical protein	178	1485	5070
CT_470	-	+	DNA repair protein RecO	243	869	3254
CT_471	-	-	hypothetical protein	200	1370	2541
CT_472	-	+	hypothetical protein	264	1581	701
CT_473	-	+	hypothetical protein	104	2095	8284
CT_474	-	-	hypothetical protein	309	1027	324
CT_475	pheT	+	phenylalanine--tRNA ligase subunit beta	790	38	401
CT_476	-	+	hypothetical protein	321	1591	1613
CT_477	ada	+	methylated-DNA protein-cysteine methyltransferase	170	974	14455
CT_478	oppC_2	-	oligopeptide ABC transporter permease	578	463	39
CT_479	oppB_2	-	oligopeptide ABC transporter permease	492	12206	4439
CT_480	oppA_4	-	oligopeptide ABC transporter substrate-binding lipoprotein	696	47	51
CT_480.1	-	-	hypothetical protein	54	559	5449
CT_481	-	-	hypothetical protein	243	2753	6767
CT_482	-	-	hypothetical protein	217	5106	18984
CT_483	-	+	hypothetical protein	121	1625	6122
CT_484	-	-	hypothetical protein	332	9078	566
CT_485	hemZ	-	ferrochetalase	314	385	1425
CT_486	fliY	-	glutamine binding protein	261	15312	3834

CT_487	yhhF	-	methylase	190	546	22419
CT_488	-	-	hypothetical protein	244	1694	4513
CT_489	glgC	-	glucose-1-phosphate adenyltransferase	441	54	578
CT_490	-	+	hypothetical protein	83	4199	14312
CT_491	rho	-	transcription termination factor	464	4391	1598
CT_492	yacE	-	dephospho-CoA kinase	202	918	2767
CT_493	polA	-	DNA polymerase I	866	722	455
CT_494	sohB	-	protease	331	22	23
CT_495	-	-	ADP/ATP translocase	540	32147	1420
CT_496	pgsA_1	-	CDP-diacylglycerol--glycerol-3-phosphate 3-phosphatidyltransferase	168	595	2441
CT_496.1	-	-	hypothetical protein	49	2188	8099
CT_497	dnaB	+	replicative DNA helicase	472	21	294
CT_498	-	+	tRNA uridine 5-carboxymethylaminomethyl modification enzyme MnmG	610	49	88
CT_499	lplA_2	+	lipoate protein ligase	233	277	900
CT_500	ndk	-	nucleoside diphosphate kinase	141	487	4687
CT_501	ruvA	-	Holliday junction ATP-dependent DNA helicase RuvA	200	11912	7137
CT_502	ruvC	-	crossover junction endodeoxyribonuclease RuvC	170	18909	10693
CT_503	-	-	hypothetical protein	184	45	731
CT_504	-	-	hypothetical protein	288	522	1875
CT_505	gapA	-	glyceraldehyde-3-phosphate dehydrogenase	334	924	3112
CT_506	rl17	-	50S ribosomal protein L17	141	211	1768
CT_507	rpoA	-	DNA-directed RNA polymerase subunit alpha	377	263	1058
CT_508	rs11	-	30S ribosomal protein S11	132	65437	15883
CT_509	rs13	-	30S ribosomal protein S13	122	945	2496
CT_510	secY	-	protein translocase subunit SecY	457	2793	634
CT_511	rl15	-	50S ribosomal protein L15	144	5523	9404
CT_512	rs5	-	30S ribosomal protein S5	165	3930	4446
CT_513	rl18	-	50S ribosomal protein L18	123	158	3812
CT_514	rl6	-	50S ribosomal protein L6	183	1193	6897

CT_515	rs8	-	30S ribosomal protein S8	133	2885	28081
CT_516	rl5	-	50S ribosomal protein L5	180	279	12158
CT_517	rl24	-	50S ribosomal protein L24	111	376	9801
CT_518	rl14	-	50S ribosomal protein L14	122	2463	17446
CT_519	rs17	-	30S ribosomal protein S17	83	456	4435
CT_520	rl29	-	50S ribosomal protein L29	72	479	4826
CT_521	rl16	-	50S ribosomal protein L16	138	959	23539
CT_522	rs3	-	30S ribosomal protein S3	224	9244	20467
CT_523	rl22	-	50S ribosomal protein L22	111	1023	17908
CT_524	rs19	-	30S ribosomal protein S19	88	209	4791
CT_525	rl2	-	50S ribosomal protein L2	284	80	833
CT_526	rl23	-	50S ribosomal protein L23	111	1676	8090
CT_527	rl4	-	50S ribosomal protein L4	222	970	11216
CT_528	rl3	-	50S ribosomal protein L3	221	6096	8670
CT_529	-	-	hypothetical protein	298	221	1177
CT_530	fmt	-	methionyl tRNAformyltransferase	316	5257	1196
CT_531	lpxA	-	acyl-ACP--UDP-N-acetylglucosamine O-acyltransferase	280	3640	2176
CT_532	fabZ	-	3-hydroxyacyl-ACP dehydratase	153	14416	1595
CT_533	lpxC	-	UDP-3-O-[3-hydroxymyristoyl] N-acetylglucosamine deacetylase	286	817	4450
CT_534	cutE	-	apolipoprotein N-acetyltransferase	542	233	173
CT_535	yciA	-	acyl-CoA thioester hydrolase	160	1203	5700
CT_536	dnaQ_2	-	DNA polymerase III subunit epsilon	250	442	6645
CT_537	yjeE	-	ATPase/kinase	157	5074	15377
CT_538	-	-	hypothetical protein	238	1066	2408
CT_539	trxA	+	thioredoxin	102	466	6230
CT_540	yibK	-	SpoU family rRNA methylase	151	1090	8288
CT_541	mip	-	peptidyl-prolyl cis-trans isomerase	243	2345	5484
CT_542	aspS	-	aspartate--tRNA(Asp/Asn) ligase	582	217	470
CT_543	hisS	-	histidine--tRNA ligase	428	184	938

CT_544	uhpC	+	hexose phosphate transporter	456	2128	721
CT_545	dnaE	+	DNA polymerase III subunit alpha	1237	45	45
CT_546	-	-	outer membrane protein	289	17169	5523
CT_547	-	+	hypothetical protein	318	2141	578
CT_548	-	+	hypothetical protein	194	3637	11452
CT_549	rsbW	+	serine/threonine kinase	146	1555	7320
CT_550	-	-	hypothetical protein	141	8701	4408
CT_551	dacC	+	D-alanine-D-alanine carboxypeptidase	343	180	226
CT_552	-	+	hypothetical protein	135	6472	6878
CT_553	fmu	+	RNA methyltransferase	325	2248	3372
CT_554	brnQ	+	branched-chain amino acid transporter	411	5199	785
CT_555	-	+	SWI/SNF family helicase	1199	29	135
CT_556	-	+	hypothetical protein	159	162	1730
CT_557	lpdA	+	dihydrolipoyl dehydrogenase	465	334	674
CT_558	lipA	+	lipoate synthetase	311	88	795
CT_559	yscJ	+	type III secretion system protein YscJ	326	1014	3093
CT_560	-	+	hypothetical protein	278	1228	3031
CT_561	yscL	+	type III secretion system protein YscL	223	749	3309
CT_562	yscR	+	type III secretion system protein YscR	306	4712	3053
CT_563	yscS	+	type III secretion system protein YscS	94	11657	45537
CT_564	yscT	+	type III secretion system protein YscT	289	469	2915
CT_565	-	-	hypothetical protein	147	3470	3325
CT_566	-	-	hypothetical protein	330	7681	2130
CT_567	-	-	hypothetical protein	174	61229	15261
CT_568	-	-	hypothetical protein	151	16310	3095
CT_569	-	-	outer membrane protein	109	1149	6552
CT_570	gspF	-	type II secretion system protein GspF	391	2918	1324
CT_571	gspE	-	type II secretion system protein GspE	501	227	64
CT_572	gspD	-	type II secretion system protein GspD	760	316	166
CT_573	-	-	hypothetical protein	409	967	1081
CT_574	pepP	-	aminopeptidase P	356	2366	2198

CT_575	mutL	-	DNA mismatch repair protein MutL	576	100	639
CT_576	lcrH_1	+	low calcium response protein H	232	79	3911
CT_577	-	+	hypothetical protein	119	31	3583
CT_578	-	+	hypothetical protein	487	1366	650
CT_579	-	+	hypothetical protein	439	5581	1036
CT_580	-	-	hypothetical protein	327	4042	979
CT_581	thrS	+	threonine--tRNA ligase	635	130	254
CT_582	minD	+	chromosome partitioning ATPase	255	6941	13664
CT_583	gp6D	+	virulence plasmid protein-like protein	263	28	378
CT_584	-	+	hypothetical protein	183	8617	21114
CT_585	trpS	+	tryptophan--tRNA ligase	346	10	74
CT_586	uvrB	+	excinuclease ABC subunit B	668	49	97
CT_587	eno	+	enolase	424	70	1335
CT_588	rbsU	+	serine phosphatase RsbU	650	261	595
CT_589	-	+	hypothetical protein	602	220	233
CT_590	-	-	hypothetical protein	954	34	439
CT_591	sdhB	-	succinate dehydrogenase iron sulfur subunit	232	2282	1685
CT_592	sdhA	-	succinate dehydrogenase flavoprotein subunit	573	95	141
CT_594	ycfH	-	PHP superfamily hydrolase	263	2221	4854
CT_595	dsbD	-	thio:disulfide Interchange protein	692	376	361
CT_596	exbB	+	polysaccharide transporter	232	14035	7894
CT_597	exbD	+	biopolymer transport protein	135	11052	22107
CT_598	-	+	hypothetical protein	235	3606	6414
CT_599	tolB	+	protein TolB	431	28	275
CT_600	pal	+	peptidoglycan-associated lipoprotein	188	6827	6382
CT_601	papQ	+	invasin repeat family phosphatase	200	1009	9219
CT_602	-	+	hypothetical protein	130	5688	7853
CT_603	ahpC	-	thio-specific antioxidant peroxidase	195	3582	3524
CT_604	groEL_2	-	chaperonin GroEL	533	112	26
CT_605	-	-	hypothetical protein	409	7091	1836
CT_606	-	+	non-canonical purine NTP pyrophosphatase	209	1197	2696

CT_606.1	-	-	hypothetical protein	79	663	9957
CT_607	ung	-	uracil DNA glycosylase	229	415	1819
CT_608	uvrD	+	DNA helicase	634	572	177
CT_609	rpoN	+	RNA polymerase sigma factor sigma-54	436	69	260
CT_610	-	-	pyrroloquinoline quinone biosynthesis protein C	231	204	1064
CT_611	-	-	hypothetical protein	243	1354	4838
CT_612	folA	-	dihydrofolate reductase	159	424	11680
CT_613	folP	-	bifunctional 2-amino-4-hydroxy-6-hydroxymethyldihydropteridine pyrophosphokinase/dihydropteroate synthase	450	7734	2461
CT_614	folX	-	dihydroneopterin aldolase	124	3959	10440
CT_615	sigA	-	RNA polymerase sigma factor SigA	571	823	727
CT_616	-	-	hypothetical protein	429	1031	663
CT_617	rs20	+	30S ribosomal protein S20	98	110	3807
CT_618	-	+	hypothetical protein	266	2166	3185
CT_619	-	-	hypothetical protein	877	553	356
CT_620	-	+	hypothetical protein	838	10	4
CT_621	-	-	hypothetical protein	832	10	155
CT_622	-	-	hypothetical protein	647	245	1991
CT_623	-	-	hypothetical protein	432	12652	4548
CT_624	mviN	-	integral membrane protein	536	313	178
CT_625	nfo	+	endonuclease IV	288	19	302
CT_626	rs4	-	30S ribosomal protein S4	209	120	3831
CT_627	yceA	+	hypothetical protein	327	8955	2918
CT_628	ispA	-	geranylgeranyl pyrophosphate synthase	291	180	873
CT_630	cpxR	-	response regulator	227	2019	2492
CT_631	-	+	hypothetical protein	84	4095	18497
CT_632	-	-	hypothetical protein	529	17	159
CT_633	hemB	+	delta-aminolevulinic acid dehydratase	338	347	2882
CT_634	-	-	Na(+)-translocating NADH-quinone reductase subunit A	465	118	390

CT_635	-	-	hypothetical protein	144	198	3045
CT_636	greA	+	transcription elongation factor GreA	715	102	62
CT_637	tyrB	+	aromatic amino acid aminotransferase	400	7914	1909
CT_639	recB	-	exodeoxyribonuclease V subunit beta	1026	2394	6302
CT_640	recC	-	exodeoxyribonuclease V subunit gamma	1004	85	82
CT_641	ygeD	+	efflux protein	559	45152	530
CT_642	-	-	hypothetical protein	271	1913	875
CT_643	topA	+	DNA topoisomerase I	857	9	34
CT_644	yohI	-	tRNA-dihydrouridine synthase	334	32	1399
CT_645	-	-	hypothetical protein	98	8040	11660
CT_646	-	+	hypothetical protein	459	193	131
CT_647	-	+	hypothetical protein	192	898	8485
CT_648	-	+	hypothetical protein	424	111	454
CT_649	ygfA	+	formyltetrahydrofolate synthetase	178	1385	9917
CT_650	recA	+	recombinase RecA	352	3698	10568
CT_651	-	+	hypothetical protein	608	78	422
CT_652	recD_2	+	exodeoxyribonuclease V subunit alpha	496	2745	1267
CT_652.1	-	+	hypothetical protein	59	110	14203
CT_653	yhbG	-	ABC transporter ATPase	239	1414	9071
CT_654	-	-	hypothetical protein	162	7973	2364
CT_655	kdsA	-	2-dehydro-3-deoxyphosphooctonate aldolase	269	73	1892
CT_656	-	+	hypothetical protein	97	727	41016
CT_657	-	+	hypothetical protein	105	587	3794
CT_658	sfhB	+	pseudouridine synthase	334	28	1554
CT_659	-	+	hypothetical protein	78	334	16055
CT_660	gyrA_2	-	DNA gyrase subunit A	490	56	252
CT_661	gyrB_2	-	DNA gyrase subunit B	605	533	892
CT_662	hemA	+	glutamyl tRNA reductase	335	21021	842
CT_663	-	+	hypothetical protein	133	733	4415
CT_664	-	+	adenylate cyclase-like protein	829	131	300
CT_665	-	+	hypothetical protein	83	112	1140

CT_666	-	+	hypothetical protein	83	394	12447
CT_667	-	+	hypothetical protein	149	875	1846
CT_668	-	+	hypothetical protein	223	315	17527
CT_669	yscN	+	type III secretion system ATPase	442	1289	1119
CT_670	-	+	hypothetical protein	168	496	4758
CT_671	-	+	hypothetical protein	283	350	636
CT_672	-	+	type III secretion system protein	373	9834	663
CT_673	pkn5	+	serine/threonine protein kinase	490	24	847
CT_674	yscC	+	type II secretion system protein YscC	921	33	60
CT_675	karG	-	arginine kinase	356	1034	2417
CT_676	-	-	hypothetical protein	173	114	2618
CT_677	rrf	-	ribosome-recycling factor	179	19294	3230
CT_678	pyrH	-	uridylate kinase	245	986	2804
CT_679	tsf	-	elongation factor TS	282	245	1014
CT_680	rs2	-	30S ribosomal protein S2	282	74	1399
CT_681	ompA	-	major outer membrane protein	393	1595	1420
CT_682	pbpB	+	transglycolase/transpeptidase	1080	249	918
CT_683	-	+	hypothetical protein	335	1289	1165
CT_684	-	+	iron-sulfur assembly protein SufB	483	9377	1359
CT_685	-	+	iron-sulfur assembly protein SufC	255	262	2308
CT_686	-	+	iron-sulfur assembly protein SufD	395	513	1032
CT_687	-	+	cysteine desulfurase-like protein	401	74	188
CT_688	parB	+	chromosome partitioning protein ParB	281	2276	2230
CT_689	dppF	-	oligopeptide ABC transporter ATPase	276	556	1226
CT_690	dppD	-	oligopeptide ABC transporter ATPase	321	710	882
CT_691	-	+	hypothetical protein	224	1553	7714
CT_692	-	+	phosphate permease	426	2440	1026
CT_693	pgk	+	phosphoglycerate kinase	403	2789	4557
CT_694	-	+	hypothetical protein	323	43	162
CT_695	-	+	hypothetical protein	398	59	38
CT_696	-	+	hypothetical protein	392	545	1909

CT_697	nth	-	endonuclease III	211	1821	9632
CT_698	-	-	tRNA modification GTPase	444	4856	1108
CT_699	psdD	+	phosphatidylserine decarboxylase	301	10640	2865
CT_700	-	+	hypothetical protein	441	594	9729
CT_701	secA_2	+	protein translocase subunit SecA	969	2360	1417
CT_702	-	-	hypothetical protein	175	3596	4021
CT_703	-	-	GTPase Der	490	44	160
CT_704	pcnB_2	-	poly A polymerase	410	873	792
CT_705	clpX	-	ATP-dependent Clp protease ATP-binding subunit ClpX	419	93	289
CT_706	clpP_2	-	ATP-dependent Clp protease proteolytic subunit	203	5646	15985
CT_707	tig	-	trigger factor	442	2860	1046
CT_708	-	+	SWF/SNF family helicase	1163	84	41
CT_709	mreB	+	cell shape determining protein	366	183	1864
CT_710	pckA	+	phosphoenolpyruvate carboxykinase	599	756	535
CT_711	-	+	hypothetical protein	767	35	424
CT_712	-	+	hypothetical protein	390	59	1130
CT_713	porB	-	outer membrane protein porB	340	4281	909
CT_714	gpdA	-	glycerol-3-phosphate dehydrogenase	334	6187	6956
CT_715	-	-	UDP-glucose pyrophosphorylase	455	4022	1342
CT_716	-	-	hypothetical protein	121	915	9804
CT_717	-	-	type III secretion system ATP synthase	434	2041	1229
CT_718	-	-	hypothetical protein	174	1398	6951
CT_719	-	-	type III secretion system protein	334	3892	4356
CT_720	-	-	NifU-like protein	260	459	3981
CT_721	yfhO_2	-	NifS-like protein	384	30	698
CT_722	pgm	-	2,3-bisphosphoglycerate-dependent phosphoglycerate mutase	226	187	1238
CT_723	yjbC	+	pseudouridine synthase	241	482	4089
CT_724	-	+	hypothetical protein	174	713	1464
CT_725	birA	+	biotin synthetase	184	3018	5662

CT_726	rodA	-	cell shape determining protein	379	33086	1264
CT_727	zntA	-	metal transport ATPase	659	1847	2521
CT_728	-	-	hypothetical protein	248	17303	1783
CT_729	serS	-	serine--tRNA ligase	428	95	20
CT_730	ribD	+	bifunctional diaminohydroxyphosphoribosyl-aminopyrimidine deaminase/5-amino-6-(5-phosphoribosylamino) uracil reductase	375	86	349
CT_731	ribA/ribB	+	bifunctional GTP cyclohydratase/ 3,4-dihydroxy-2-butanone-4-phosphate synthase	424	3552	948
CT_732	ribE	+	7-dimethyl-8-ribityllumazine synthase	157	7710	17778
CT_733	-	-	hypothetical protein	448	191	3892
CT_734	-	+	lipoprotein	221	601	2431
CT_735	dagA_2	+	D-alanine/glycine permease	455	5530	1193
CT_736	ybcL	+	hypothetical protein	150	790	2273
CT_737	-	-	hypothetical protein	219	2902	3449
CT_738	yycJ	-	metal dependent hydrolase	262	476	1551
CT_739	ftsK	-	DNA translocase FtsK	799	128	66
CT_740	dmpP	-	Na(+)-translocating NADH-quinone reductase subunit F	431	3812	2007
CT_741	-	-	hypothetical protein	114	2449	14323
CT_742	ygcA	-	rRNA methyltransferse	396	27	1007
CT_743	hctA	-	histone-like protein	125	627	4477
CT_744	-	+	hypothetical protein	821	62	304
CT_745	hemG	-	protoporphyrinogen oxidase	424	331	589
CT_746	hemN_2	-	cproporphyrinogen III oxidase	457	322	1742
CT_747	hemE	-	uroporphyrinogen decarboxylase	336	420	2057
CT_748	mfd	-	transcription-repair coupling factor	1079	7	489
CT_749	alaS	-	alanine--tRNA ligase	875	9	12
CT_750	tktB	+	transketolase	666	263	762
CT_751	amn	-	AMP nucleosidase	289	3314	6238
CT_752	efp_2	+	elongation factor P	190	3858	1100

CT_753	-	-	hypothetical protein	74	25	1265
CT_754	icc	-	phosphohydrolase	290	14	595
CT_755	groEL_3	-	chaperonin GroEL	512	2513	554
CT_756	murF	+	UDP-N-acetylmuramoylalanyl-D-glutamyl-2,6-diaminoligase	450	1840	1654
CT_757	mraY	+	phospho-N-acetylmuramoyl-pentapeptide-transferase	336	2687	371
CT_758	murD	+	UDP-N-acetylmuramoylalanine-D-glutamate ligase	416	44	298
CT_759	nlpD	+	muramidase	245	389	2881
CT_760	ftsW	+	cell division protein FtsW	385	10204	899
CT_761	murG	+	UDP-N-acetylglucosamine--N-acetylmuramyl-(pentapeptide) pyrophosphoryl-undecaprenol N-acetylglucosamine transferase	352	2939	3749
CT_762	murC/ddlA	+	bifunctional UDP-N-acetylmuramate-alanine ligase/D-alanine-D-alanine ligase	803	69	284
CT_763	-	-	hypothetical protein	139	378	6554
CT_764	-	-	hypothetical protein	268	689	1504
CT_765	rsbV_2	+	anti-sigma factor antagonist	110	634	2203
CT_766	miaA	+	tRNA dimethylallyltransferase	339	114	1379
CT_767	-	-	Fe-S cluster oxidoreduase	350	24	641
CT_768	-	+	hypothetical protein	562	571	338
CT_769	ybeB	+	ribosome silencing factor RsfS	119	1317	12009
CT_770	fabF	+	3-oxoacyl-ACP synthase	418	4532	416
CT_771	-	+	hydrolase/phosphatase	150	389	2467
CT_772	ppa	-	inorganic pyrophosphatase	209	231	2026
CT_773	ldh	+	leucine dehydrogenase	346	4557	1138
CT_774	cysQ	-	3'(2')5'-bisphosphate nucleotidase CysQ	342	23	946
CT_775	-	+	sn=glycerol 3-phosphate acyltransferase	253	1160	5917
CT_776	aas	+	acylglycerophosphoethanolamine acyltransferase	537	48	645
CT_777	bioF	+	8-amino-7-oxononanoate synthase	377	856	1214

CT_778	priA	-	primosomal protein N'	753	297	398
CT_779	-	-	hypothetical protein	229	178	16356
CT_780	-	+	thioredoxin disulfide isomerase	164	329	1267
CT_781	lysS	+	lysine--tRNA ligase	526	25	206
CT_782	cysS	-	cysteine--tRNA ligase	497	13	35
CT_783	-	+	disulfide bond isomerase	349	136	4675
CT_784	rnpA	-	ribonuclease P protein component	120	187	5751
CT_785	rl34	-	50S ribosomal protein L34	45	58	3827
CT_786	rl36	+	50S ribosomal protein L36	45	50	4411
CT_787	rs14	+	30S ribosomal protein S14	101	172	26422
CT_788	-	-	leader (60) peptide	166	8223	10688
CT_789	-	-	hypothetical protein	83	7565	10083
CT_790	-	+	hypothetical protein	164	5634	12649
CT_791	uvrC	-	excinuclease ABC subunit C	598	40	24
CT_792	mutS	-	DNA mismatch repair protein MutS	820	51	155
CT_793	-	+	hypothetical protein	89	590	12859
CT_794	dnaG	+	DNA primase	595	75	12
CT_794.1	-	+	hypothetical protein	95	811	5258
CT_795	-	+	hypothetical protein	163	1530	7865
CT_796	glyQ	-	glycine--tRNA ligase	1003	456	2498
CT_797	pgsA_2	+	CDP-diacylglycerol--glycerol-3-phosphate 3-phosphatidyltransferase	202	46921	4273
CT_798	glgA	-	glycogen synthase	474	487	1319
CT_799	-	+	50S ribosomal protein L25	185	2145	2451
CT_800	pth	+	peptidyl tRNA hydrolase	179	2669	4151
CT_801	rs6	+	30S ribosomal protein S6	112	3651	17756
CT_802	rs18	+	30S ribosomal protein S18	81	75	937
CT_803	rl9	+	50S ribosomal protein L9	167	5228	11396
CT_804	ychB	+	4-diphosphocytidyl-2-C-methyl-D-erythritol kinase	288	245	2147
CT_805	-	-	hypothetical protein	450	183	324
CT_806	ptr	-	insulinase family protease III	956	5649	2608

CT_807	plsB	-	glycerol-3-phosphate acyltransferase	331	611	2099
CT_808	cafE	-	Rne/Rng family ribonuclease	512	4627	1219
CT_809	-	+	hypothetical protein	103	11141	5304
CT_810	rl32	+	50S ribosomal protein L32	59	596	16629
CT_811	plsX	+	phosphate acyltransferase	321	495	3382
CT_812	pmpD	+	outer membrane protein PmpD	1531	355	2644
CT_813	-	+	hypothetical protein	264	7653	4637
CT_814	-	-	hypothetical protein	133	1263	5580
CT_814.1	-	-	hypothetical protein	120	1448	7093
CT_815	mrsA_2	+	phosphoglucosamine mutase	458	93	252
CT_816	glmS	+	glucosamine-fructose-6-phosphate aminotransferase	606	441	2362
CT_817	tyrP_1	+	tyrosine transporter permease	398	2993	448
CT_818	tyrP_2	+	tyrosine transporter permease	397	199	583
CT_819	yccA	+	hypothetical protein	238	4181	7642
CT_820	ftsY	-	signal recognition particle receptor FtsY	284	95	517
CT_821	sucC	+	succinyl-CoA ligase subunit beta	386	35	267
CT_822	sucD	+	succinyl-CoA ligase subunit alpha	291	1088	1700
CT_823	htrA	+	DO serine protease	497	16	171
CT_824	-	+	zinc metalloprotease	974	21	81
CT_825	-	-	DNA recombination protein RmuC	427	191	505
CT_826	pssA	-	CDP-diacylglycerol-serine-O-phosphatidyltransferase	263	13426	695
CT_827	nrdA	+	ribonucleoside-diphosphate reductase subunit alpha	1047	61	128
CT_828	nrdB	+	ribonucleoside-diphosphate reductase subunit beta	346	212	2626
CT_829	yggH	+	rRNA methylase	187	389	2284
CT_830	ytgB_2	+	tRNA (guanine-N(7)-)-methyltransferase	194	1389	3124
CT_831	murB	-	UDP-N-acetylenolpyruvoylglucosamine reductase	291	812	898
CT_832	nusB	-	transcription termination factor	168	644	5822
CT_833	infC	+	translation initiation factor IF-3	175	580	2104
CT_834	rl35	+	50S ribosomal protein L35	64	215	2128
CT_835	rl20	+	50S ribosomal protein L20	123	3526	20727
CT_836	pheS	+	phenylalanine--tRNA ligase subunit alpha	342	892	2475

CT_837	-	+	hypothetical protein	658	64	181
CT_838	-	-	hypothetical protein	366	51	1174
CT_839	-	-	hypothetical protein	354	1814	904
CT_840	mesJ	+	tRNA(Ile)-lysidine synthase	321	1491	709
CT_841	ftsH	+	ATP-dependent zinc metalloprotease FtsH	913	2027	5945
CT_842	pnp	-	polyribonucleotide nucleotidyltransferase	695	20	53
CT_843	rs15	-	30S ribosomal protein S15	89	115	10627
CT_844	yfhC	+	tRNA-specific adenosine deaminase	163	484	5025
CT_845	-	+	hypothetical protein	92	89	2169
CT_846	-	-	hypothetical protein	234	5533	5584
CT_847	-	-	hypothetical protein	172	57632	7699
CT_848	-	-	hypothetical protein	168	8492	5242
CT_849	-	-	hypothetical protein	159	485	5661
CT_849.1	-	+	hypothetical protein	62	395	12179
CT_850	-	+	hypothetical protein	405	288	765
CT_851	map	-	methionine aminopeptidase	291	113	1890
CT_852	-	-	membrane protein	204	24096	8822
CT_853	-	-	hypothetical protein	199	17479	3395
CT_854	-	-	ABC transporter permease/substrate-binding protein	589	204	2237
CT_855	fumC	+	fumarate hydratase	463	241	1024
CT_856	ychM	-	sulfate transporter	567	14	45
CT_857	-	+	Na+/H+ antiporter	421	296	328
CT_858	-	+	hypothetical protein	601	3093	517
CT_859	lytB	+	4-hydroxy-3-methylbut-2-enyl diphosphate reductase	307	335	570
CT_860	-	-	hypothetical protein	493	12	36
CT_861	-	-	hypothetical protein	506	34	332
CT_862	lcrH_2	-	low calcium response protein H	198	5810	15214
CT_863	-	-	hypothetical protein	482	96	286
CT_864	xerD	-	tyrosine recombinase XerD	300	1629	3483
CT_865	-	+	hypothetical protein	329	143	732
CT_866	glgB	+	1,4-alpha-glucan branching enzyme	738	27	1606

CT_867	-	-	deubiquitinase/deneddylase Dub2	339	9533	2396
CT_868	-	-	deubiquitinase/deneddylase Dub1	418	96	221
CT_869	pmpE	-	outer membrane protein PmpE	964	1528	2525
CT_870	pmpF	-	outer membrane protein PmpF	1034	193	1000
CT_871	pmpG	+	outer membrane protein PmpG	1013	10	77
CT_872	pmpH	+	outer membrane protein PmpH	1016	268	206
CT_873	-	+	hypothetical protein	105	16270	14296
CT_874	pmpI	-	outer membrane protein PmpI	878	34	193
CT_875	-	+	hypothetical protein	591	819	2267
***Chlamydia trachomatis* plasmid: 8 proteins**						
pCHL1p3	pCHL1p3		pGP1	451	6184	687
pCHL1p4	pCHL1p4		pGP2	354	115	88
pCHL1p5	pCHL1p5		pGP3	264	1917	4380
pCHL1p6	pCHL1p6		pGP4	102	171	14200
pCHL1p7	pCHL1p7		pGP5	243	48	2322
pCHL1p8	pCHL1p8		pGP6	247	511	2584
pCHL1p1	pCHL1p1		pGP7	305	1936	2853
pCHL1p2	pCHL1p2		pGP8	330	51864	4213

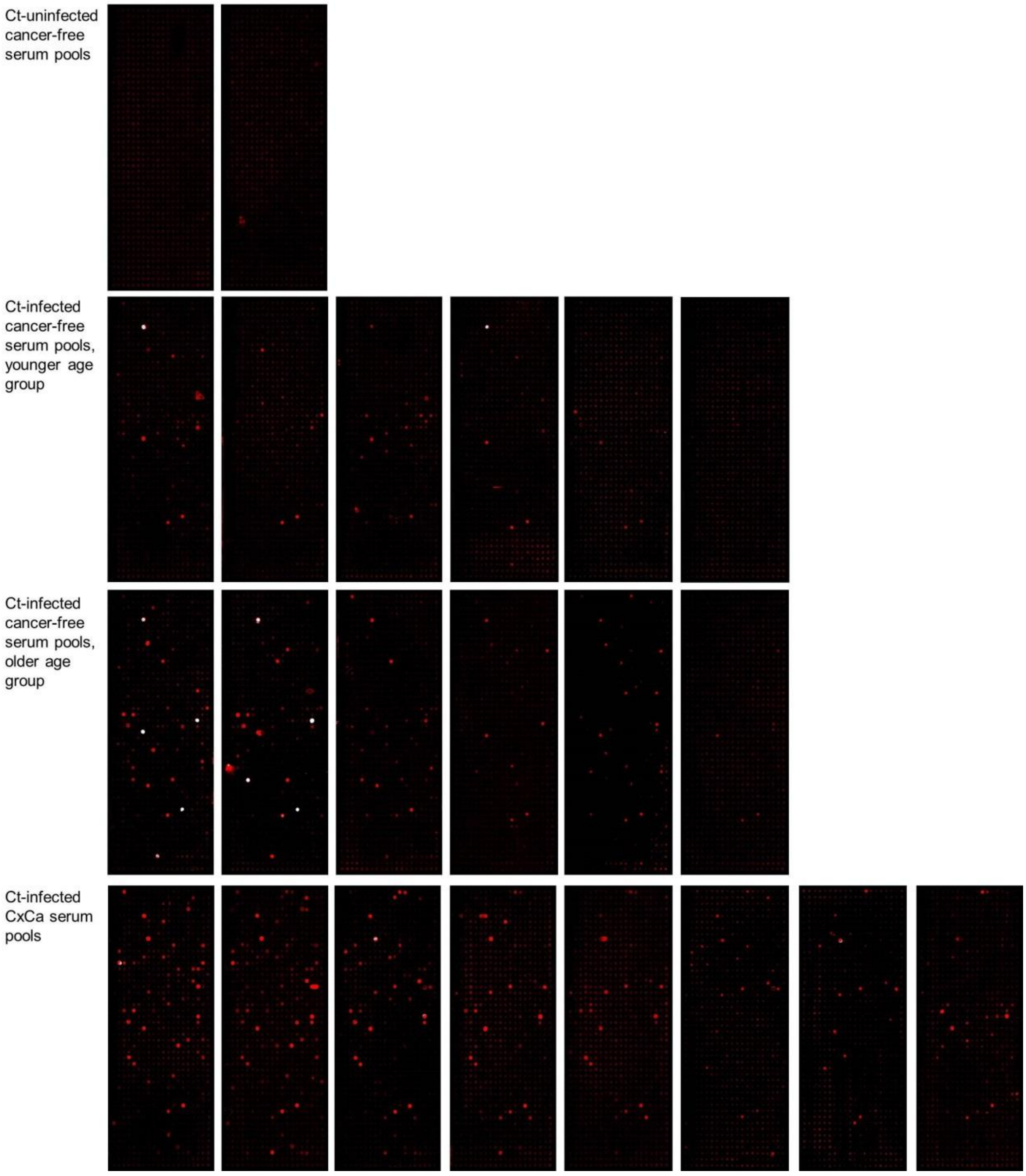

Supplementary figure S2: Proteome Immunoassays (PIAs) using pools of five sera each. In total, 897 Ct proteins and one Cp protein were spotted on each array. No signal was obtained with pools from Ct-uninfected women (n=2). PIAs with pools from Ct-infected cancer-free women (younger and older age group, n=6 each) as well as pools from Ct-infected CxCa patients revealed increasing signal density.

Supplementary table S3: Comparison of the newly identified Ct antigens with the already known immunogenic protein pGP3. In total 985 mongolian sera were analyzed using multiplex serology. Antibodies against newly identified antigens and pGP3 were measured and compared. CI: confidence interval

		pGP3		Kappa	95 % CI
		1	0		
CT_142	1	644	16	0.83	0.76 - 0.89
	0	57	268		
CT_143	1	659	73	0.71	0.64 - 0.77
	0	42	211		
CT_798	1	686	102	0.68	0.68 - 0.74
	0	15	182		
CT_813	1	630	40	0.73	0.67 - 0.80
	0	71	244		
